# Self-evaluation of social-rank in socially anxious individuals associates with enhanced striatal reward function

**DOI:** 10.1017/S0033291722001453

**Published:** 2023-07

**Authors:** Ofir Shany, Netta Dunsky, Gadi Gilam, Ayam Greental, Eva Gilboa-Schechtman, Talma Hendler

**Affiliations:** 1 School of Psychological Sciences, Tel-Aviv University, Tel-Aviv, Israel; 2 Sagol Brain Institute, Tel-Aviv Sourasky Medical Center, Tel-Aviv, Israel; 3 Sagol School of Neuroscience, Tel-Aviv University, Tel-Aviv, Israel; 4 Faculty of Dental Medicine, The Institute of Biomedical and Oral Research, Hebrew University of Jerusalem, Jerusalem, Israel; 5 Department of Psychology and the Gonda Brain Science Center, Bar-Ilan University, Ramat-Gan, Israel; 6 Sackler School of Medicine, Tel-Aviv University, Tel-Aviv, Israel

**Keywords:** Emotion, fMRI, Reward, Self-evaluation, Social anxiety

## Abstract

**Background:**

Negative self-views, especially in the domain of power (i.e. social-rank), characterize social anxiety (SA). Neuroimaging studies on self-evaluations in SA have mainly focused on subcortical threat processing systems. Yet, self-evaluation may concurrently invoke diverse affective processing, as motivational systems related to desired self-views may also be activated. To investigate the conflictual nature that may accompany self-evaluation of certain social domains in SA, we examined brain activity related to both threat and reward processing.

**Methods:**

Participants (*N* = 74) differing in self-reported SA-severity underwent fMRI while completing a self-evaluation task, wherein they judged the self-descriptiveness of high- *v.* low-intensity traits in the domains of power and affiliation (i.e. social connectedness). Participants also completed two auxiliary fMRI tasks designated to evoke reward- and threat-related activations in the ventral striatum (VS) and amygdala, respectively. We hypothesized that self-evaluations in SA, particularly in the domain of power, involve aberrant brain activity related to both threat and reward processing.

**Results:**

SA-severity was more negatively associated with power than with affiliation self-evaluations. During self-evaluative judgment of high-power (e.g. dominant), SA-severity associated with increased activity in the VS and ventromedial prefrontal cortex. Moreover, SA-severity correlated with higher similarity between brain activity patterns activated by high-power traits and patterns activated by incentive salience (i.e. reward anticipation) in the VS during the reward task.

**Conclusions:**

Our findings indicate that self-evaluation of high-power in SA involves excessive striatal reward-related activation, and pinpoint the downregulation of VS-VMPFC activity within such self-evaluative context as a potential neural outcome for therapeutic interventions.

## Introduction

Social anxiety (SA) is a widespread affective disturbance that is marked by debilitating fear and avoidance of social situations (Leichsenring & Leweke, 2017). SA is associated with erosion of positivity in self-evaluations with regards to specific domains (Gilboa-Schechtman, Keshet, Peschard, & Azoulay, 2020). Therefore, it is crucial to elucidate the affective processes that accompany non-positive self-evaluations in SA. Based on social-cognitive perspectives on self-processing and clinical theories of SA, here we test the possibility that specific content of self-views may expose relations between SA and affective brain activity during non-positive self-evaluation.

Negative self-evaluation in SA may center around specific social domains (Gilboa-Schechtman et al., 2020; Moscovitch, 2009). One conceptualization of such domains derives from the evolutionary perspective on SA (Gilbert, 2001). This viewpoint suggests that high-SA individuals are sensitive to cues denoting social *power* (aka social-rank, status, dominance), and perceive themselves as possessing low power. Moreover, excessive attunement to power-related cues often comes at the expense of attentiveness towards signals of *affiliation* (i.e. social connectedness). Respectively, high SA individuals assign enhanced significance to power-related traits and evaluate themselves more negatively on power-related *v.* affiliative traits (Aderka, Weisman, Shahar, & Gilboa-Schechtman, 2009; Berger, Keshet, & Gilboa-Schechtman, 2017; Dijk, van Emmerik, & Grasman, 2018; Gilboa-Schechtman et al., 2017; Kashdan, Elhai, & Breen, 2008; Roberts, Hart, Coroiu, & Heimberg, 2011; Weisman, Aderka, Marom, Hermesh, & Gilboa-Schechtman, 2011).

Two prominent social-cognitive theories have described possible connections between self-evaluations and affective processing. The theory of ‘possible selves’ (Markus & Nurius, 1986) states that mental representations of what individuals could hopefully become, or of what they are afraid of becoming, are cognitive manifestations of underlying desires or threats, respectively. The ‘self-discrepancy’ theory (Higgins, 1987) posits that incompatibility between desired and actual self-views may elicit negative emotions and dampen positive emotions. These theories both suggest that pondering desired (but not actual) and non-desired traits, and evaluating these traits against the current self, mobilizes affective processes that are likely mediated through dedicated neural systems. Specifically, self-evaluation may encompass signaling valuable self-concepts, a process that is mediated in part through the brain's reward circuit (Bartra, McGuire, & Kable, 2013; D'Argembeau et al., 2012). Furthermore, the affirmation of positive or negative qualities of the self, may arise pleasant and unpleasant feelings that scale with reward- and threat-related brain activity (Eisenberger, Inagaki, Muscatell, Haltom, & Leary, 2011; Izuma, Kennedy, Fitzjohn, Sedikides, & Shibata, 2018). High-SA individuals view power-related traits as important to their self-concept, yet they are largely discrepant from their actual self-view (Gilboa-Schechtman, Galili, Sahar, & Amir, 2014; Kashdan et al., 2008; Roberts et al., 2011). Thus, evaluation of the self in terms of power may concurrently involve altered activations in both reward- and threat-related neural circuitries in SA. Neuroimaging provides a powerful means for associating affective neural processing with self-evaluation.

Hitherto, neuroimaging studies have associated self-referential processing with cortical midline components of the default-mode network (DMN). These include dorsal and ventral portions of the medial prefrontal cortex (MPFC), as well as the precuneus and posterior cingulate cortex (PCC). The amalgamation of self-referential processing with affect likely involves subcortical regions. These include the amygdala, which is implicated in negative affective processing, including threat to the integrity of one's self-view (Frewen, Lundberg, Brimson-Théberge, & Théberge, 2013; Hughes & Beer, 2013); and the VS – a major node of the mesolimbic reward system. The VS mediates processes such as incentive salience (i.e. signaling motivationally significant stimuli); and reward responsiveness (i.e. consuming rewarding outcomes) (Bartra et al., 2013; Liu, Hairston, Schrier, & Fan, 2011). In context of self-evaluation, VS activation has been linked to processing important self-related content such as one's reputation (Izuma, Saito, & Sadato, 2010), and to affirming positive qualities of oneself (Chavez & Heatherton, 2014; Izuma et al., 2018). Given the involvement of amygdala and VS in self-evaluation, an examination of self-evaluation in SA should consider both of these affect-related neural regions.

In clinically diagnosed SA, self-referential processing increases activation in midline DMN structures and in the amygdala relative to healthy controls (Blair et al., 2008, 2011; Dixon et al., 2020; Goldin & Gross, 2010; Goldin, Manber-Ball, Werner, Heimberg, & Gross, 2009b). Moreover, while increased MPFC activity is associated with tracking the self-descriptiveness of traits (D'Argembeau, 2013; Moran, Macrae, Heatherton, Wyland, & Kelley, 2006), in SA it has been implicated in processing both negative and positive (and thus probably less self-descriptive) self-related information such as traits and social feedbacks (Goldin, Ramel, & Gross, 2009a; Heitmann et al., 2014; Peterburs, Sandrock, Miltner, & Straube, 2016). These findings concord with the enhanced self-focused attention and negative affect that characterize SA (Norton & Abbott, 2016). Yet, these studies mostly utilized personalized negative self-beliefs or valenced traits without specifying their social domain. Moreover, while these studies often focused on threat-related amygdala activity, engaging reward-related activation in the VS may also be crucial for positive self-evaluation as discussed above. Several studies show aberrant VS functionality in SA, including increased response to interpersonal situations and threatening social cues (Finlayson-Short, Harrison, & Davey, 2021; Klumpp, Angstadt, & Phan, 2012), and reduced response to social rewards (Becker, Simon, Miltner, & Straube, 2017; Schultz et al., 2019). However, the role of reward-related VS activity in poor self-evaluation in SA remains unclear. Moreover, the amygdala and VS are not exclusively dedicated to the processing of negative and positive valenced information, respectively. Rather, both regions are associated with diverse affective and motivational processes (Bartra et al., 2013; Putnam & Gothard, 2019). Thus, *which* affective processes are actually mediated by these subcortical regions during biased self-evaluation in SA demands clarification.

The current study aimed to concurrently explore reward- and threat-related brain activations that accompany self-evaluative processing in SA, while taking into account specific content dimensions of self descriptions – namely power and affiliation. Providing a more nuanced understanding of the content of concerns in SA and their underlying neural mechanisms, could inform both cognitive-behavioral- and neuromodulation-based therapeutic approaches to SA. To this end, participants ranging in SA-severity completed a self-referential encoding task (SRET) during an fMRI scan. Participants were requested to decide on the self-descriptiveness of traits derived from domains of power and affiliation. Traits differed in their denomination of high and low end of the power and affiliation continua with some traits denoting high end of the continuum (e.g. assertive, warm) and some low end of the continua (weak, hostile). To characterize the affective processes that are mediated by subcortical regions during the SRET, participants completed two additional auxiliary fMRI tasks that are known to evoke VS and amygdala activity in response to reward and threat, respectively. These included a monetary reward task, which probed participants' anticipation (i.e. incentive salience phase) and consumption (i.e. reward responsiveness phase) of monetary prizes; and a threat task, wherein participants executed a simple matching task on angry and fearful faces.

Two hypotheses were tested. First, we expected SA-severity to be more negatively associated with power than with affiliation self-evaluations (Gilboa-Schechtman et al., 2020). We hypothesized that this pattern would manifest at the neural level, such that SA-severity will be associated with greater activity in midline DMN nodes (MPFC, precuneus-PCC) and in the amygdala (Brühl, Delsignore, Komossa, & Weidt, 2014; Yoon, Seo, Kim, & Choo, 2019), during the self-evaluation of power (*v.* affiliation) traits. Second, we hypothesized that reflecting on power (*v.* affiliation) in higher SA-severity would associate with reward-related VS activity, and our hypothesis regarding this matter was two-sided. On the one hand, insufficiently positive self-evaluation in terms of power in higher SA-severity may be accompanied by *diminished* VS activity. On the other hand, SA is associated with assignment of high value to power-related traits (Kashdan et al., 2008; Roberts et al., 2011), which may be reflected by *increased* VS activity (Bartra et al., 2013). Since reward-related effects might manifest particularly with regards to more positive traits (Chavez & Heatherton, 2014) such as the high-intensity traits in this study (see online Supplementary Methods), we also examined whether brain activation in the SRET was affected by the interaction of SA-severity with both the social domain and intensity of traits. Lastly, in order to better characterize affective processes that were possibly engaged during self-evaluation of power in SA, we implemented a neural representational similarity analysis between the SRET and the auxiliary tasks. This analytical approach can reveal similarities in how the brain encodes affective and cognitive processes across tasks and mental states. We reasoned that greater neural similarity between the SRET and the reward and threat tasks in a given brain region, would provide evidence that a region mediated the latter affective processes at the time of self-evaluation (Axelrod, Rees, & Bar, 2017; Chavez, Heatherton, & Wagner, 2017; Puccetti et al., 2021). Thus, in an exploratory analysis that followed the brain activation analysis, we examined the correlation of SA with between-task similarity indices in regions whose activation was modulated as a function of SA during self-evaluation of power in the SRET.

## Methods

### Participants

We recruited seventy-four participants (M_age_ ± s.d.: 25.11 ± 3.03 years, 44 females) to this experiment following an online screening procedure (*n* = 845; see online Supplementary Methods and Fig. S1), whose purpose was to assure the recruitment of participants ranging in SA-severity as indicated by the Liebowitz Social Anxiety Scale (LSAS) (Liebowitz, 1987). The sampling strategy was a convenience/availability sampling, meaning that participants signed up to participate and were included if they fulfilled the inclusion criteria (detailed in online Supplementary Methods). Moreover, we did not conduct any formal power analysis to determine the fMRI sample size, but it is consistent with literature recommendations (Durnez et al., 2016; Turner, Paul, Miller, & Barbey, 2018) and with similar recent fMRI studies that focused on brain-behavior correlations (Will et al., 2020; Yoon, Somerville, & Kim, 2018). All participants completed the SRET; 71 completed the threat task (M_age_ ± s.d.: 25.10 ± 3.07 years, 42 females); and 54 participants completed the reward task (M_age_ ± s.d.: 25.15 ± 3.11 years, 33 females). Note that the first 20 participants completed a different reward task that involved risk taking in a social context, and we decided to replace it with a simpler and shorter task. Three/two/four participants were excluded from the fMRI analysis of the SRET/reward/threat tasks, respectively, due to technical issues and exaggerated head movement (see online Supplementary Methods). Thus, fMRI analysis was performed on 71 participants in the SRET (M_age_ ± s.d.: 25 ± 2.96 years, 43 females); 52 participants in the reward task (M_age_ ± s.d.: 25.19 ± 3.1 years, 31 females); and 67 participants in the threat task (M_age_ ± s.d.: 25 ± 2.98 years, 41 females).

### Procedure overview

At 1–2 days prior to the experiment, participants filled the LSAS questionnaire again via the *qualtrics* software. On the experimental day, participants signed an informed consent and practiced the three fMRI tasks (15–20 min). Next, they entered the MRI scanner, and completed an anatomical scan and the SRET (2 runs, ~5 min each), reward (2 runs, ~5 min each) and threat tasks (~7 min). To control for potential effects of reward on subsequent threat processing or vice versa, presentation order of the reward and threat tasks was counter-balanced across participants.

### Liebowitz Social Anxiety Scale (LSAS)

The LSAS is a 24-item scale measuring fear and avoidance of social situations over the past week. Each item is rated on two 4-point Likert scales that measure fear and avoidance regarding the item, from 0 (never) to 3 (severe). SA ranges in severity, but even below-diagnostic levels are associated with reduced well-being (Fehm, Beesdo, Jacobi, & Fiedler, 2008) and impairments in work performance and social relationships, which are akin to those evident in full-blown social anxiety disorder (SAD) (Wittchen, Fuetsch, Sonntag, Müller, & Liebowitz, 2000). Respectively, dimensional approaches to SAD suggest that its diagnosis and clinical outcomes are best explained by viewing this condition as existing along a continuum that ranges from subclinical levels and up to full syndrome levels, rather than as a dichotomous condition that is either present or absent (Ruscio, 2010). Crucially, dimensional approaches advocate for examination of subclinical levels of SA, as this may enhance accurate prognosis by identifying risk factors of this disorder (e.g. genetic), as well as guide its effective treatment (Ruscio, 2010). Therefore, we aimed to sample SA in a dimensional manner (Hyett & McEvoy, 2018) that would span from low levels of SA (LSAS ⩽ 30) and up to clinical levels that characterize SAD (LSAS ⩾ 50) and generalized SAD [LSAS ⩾ 60; (Mennin et al., 2002; Rytwinski et al., 2009). Indeed, our sample included a wide range of SA-severity (LSAS scores at screening ranged between 8–109; *M* ± s.d.: 51.35 ± 23.07). Adjacent to the experimental day (i.e. during which participants filled the LSAS questionnaire for the second time), LSAS scores ranged between 2–108 (*M* ± s.d.: 51.15 ± 26.12; see LSAS distributions in online Supplementary Fig. S1 and Supplementary Methods for more details). The correlation between the two LSAS measurements was high (*r*(72) = 0.86, *p* < 0.001), and for the analyzes we used LSAS scores obtained on the experimental day.

### fMRI tasks

#### Self-Referential Encoding Task (SRET)

Neurobehavioral processing of self-views was assessed with a modified version of the SRET (Goldin, Ziv, Jazaieri, & Gross, 2012). Throughout the task, participants were exposed to the same set of traits twice. On each presentation they either judged if the traits were self-descriptive on ‘self’ blocks; or determined whether the first 2 letters of the trait were presented in the alphabetical order on ‘control’ blocks (Axelrod et al., 2017). The stimuli consisted of 64 social trait adjectives (online Supplementary Table S1), which were classified into a 2 × 2 within-subject factorial design according to their domain (power *v.* affiliation) and intensity (high *v.* low loading of the traits on the relevant dimensions). Domain and intensity of stimuli were validated in an independent behavioral experiment (see online Supplementary Methods and Tables S1 and S2).

**Fig. 1.**
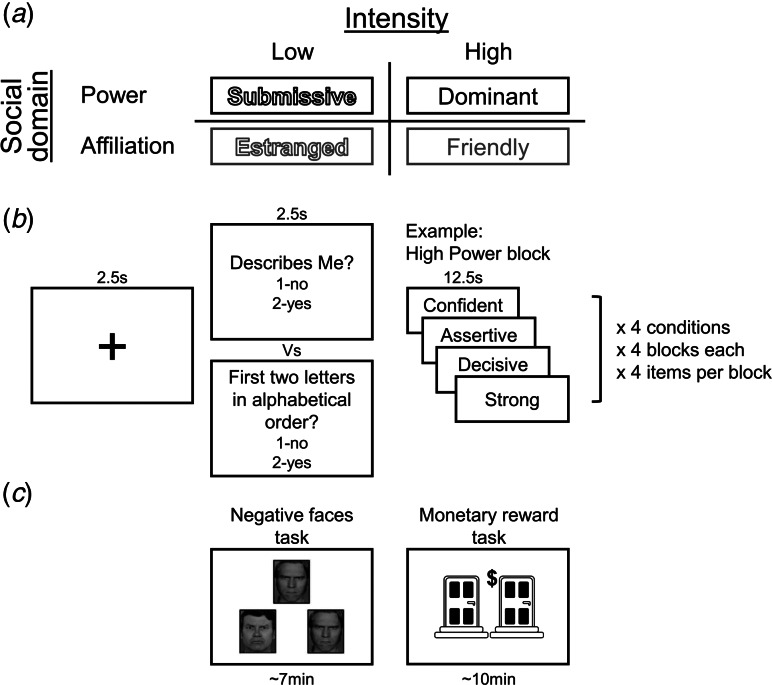
Self-referential encoding task (SRET) and experimental design. (a). The traits in the SRET were classified into four experimental conditions according to their social domain (power v. affiliation; colored in black v. gray, respectively) and intensity (low v. high; without or with color fill, respectively), thus yielding a 2 × 2 within-subject factorial design. (b). Example of an experimental block in the high-power condition. (c). Following the SRET, the participants completed two auxiliary fMRI tasks assessing brain activity in response to reward- and threat-related processes. In the threat task, participants performed a simple matching task on negative facial expressions (angry and fearful) or geometrical shapes (control condition). On each block, participants were asked to select one of two faces/shapes (located at the bottom right or bottom left of the screen) that matched the target face/shape (located at the top of the screen). In the monetary reward task, on each trial participants were asked to select one of two doors, which, following an anticipatory phase, led to either monetary prizes or losses. More details on these tasks are presented in the online Supplementary Methods and in Figs S2 and S3. Note that administration order of the two auxiliary fMRI tasks was counter-balanced across participants.

The SRET was conducted using an fMRI block design (Fig. 1). Each block initiated with a fixation (2.5s) that was followed by a task instruction screen (2.5s). Afterwards, a set of 4 traits pertaining to one of the experimental conditions was presented (12.5s; 3.125s per trait). Participants used an MRI-compatible response box to provide yes/no answers while each trait appeared. The task was performed in 2 separate runs (~5 min each), and each run contained 16 blocks and started with a 22.5s long fixation. Stimulus order included a pseudo-random block sequence with no more than two consecutive blocks of the same condition and a random sequence of words within each block. Presentation order of conditions was counterbalanced between participants, by using four different task versions. The experiment was programmed and presented in PsychoPy (version 1.85).

#### Auxiliary fMRI tasks for characterizing basic affective processes

The auxiliary tasks' structures are fully detailed in the online Supplementary Methods and Figs S2 and S3. Reward-related processes in the VS were probed via a gambling task (Carlson, Foti, Mujica-Parodi, Harmon-Jones, & Hajcak, 2011). On each trial of this event-related paradigm, participants had to select one of two doors (2.5s), which, following an anticipatory phase (2.5s), led to either monetary prizes (+5 NIS) or losses (−2.5 NIS) that were signified by an upward-pointing green arrow or a downward-pointing red arrow, respectively (1s). Unknown to participants, the task included an equal amount of predetermined wins and losses (30 trials each) that appeared in a fixed order. Stimulation of threat-related activation in the amygdala was probed through the emotional faces matching task (Hariri, Tessitore, Mattay, Fera, & Weinberger, 2002; Scult, Knodt, Radtke, Brigidi, & Hariri, 2019). In this block-design task, participants performed either a face-matching task on emotional facial expressions (angry, fearful, surprised, and neutral, presented on 4 different blocks) or a similar sensorimotor control task on geometrical shapes (5 blocks). Specifically, participants were instructed to select one of two faces/shapes (located at the bottom right or bottom left of the screen) that matched the target face/shape (located at the top of the screen). Faces block lasted 48s and shapes blocks lasted 36s. Each block consisted of 6 presentations of faces/shapes trios the lasted 4s, interleaved with fixations. Here we analyzed only angry and fearful faces, as they convey negative affect directly.

### Behavioral data analysis

#### Interaction of SA with endorsement of power *v.* affiliation traits

To examine the endorsement of traits in the SRET, we first computed the percentage of traits that participants endorsed as self-descriptive per condition. Note that due to a technical error that occurred in 40 participants in the high-power condition, one block was removed from each condition in all analyses (see online Supplementary Methods). To examine our hypothesis that SA-severity is negatively associated with endorsement of power traits above and beyond the association with affiliation traits, we tested the interaction between LSAS scores and within-subject categorical factors (domain and intensity) in predicting endorsement of traits. We conducted a linear moderated regression analysis using the *peqoud* package in *R* (https://CRAN.R-project.org/package=pequod), whose model included predictors for all main and interaction effects. Endorsement percentages of low-intensity traits were reverse-scored by subtracting them from 100, such that they would represent higher self-descriptiveness within their social domain. Interaction effects involving LSAS scores were inspected by computing simple slopes and testing their differences using the *peqoud* package as well (Dawson & Richter, 2006).

### Functional Magnetic Resonance Imaging (fMRI)

#### Imaging data acquisition

See online Supplementary Methods.

#### Imaging data preprocessing

Imaging data were preprocessed with *fMRIPrep* (Esteban et al., 2019). Details are provided in the online Supplementary Methods. Note that data of two/four participants were excluded from the analysis of the reward/threat tasks, respectively, due to head movements exceeding 3 mm.

#### Analysis of fMRI data from the SRET

Statistical analysis of the fMRI data was conducted with Statistical Parametric Mapping software (SPM12; http://www.fil.ion.ucl.ac.uk/spm). The 1st-level General Linear Model (GLM) of the task included 10 predictors: (1–8) predictors for the 8 task conditions (event duration = 12.5s); (9–10) predictors for self and control blocks that were excluded from the analysis (see online Supplementary Methods). All predictors were convolved with a canonical hemodynamic response function, and data were subjected to SPM12 default high-pass filter cutoff (128s). Confound regressors were added to the 1st-level model as well (see online Supplementary Methods). We then computed linear contrasts for the effects of social domain and for the intensity by social domain interaction for each participant. Note that within these contrasts, we also subtracted brain activity measured during the control condition blocks from their corresponding self condition blocks (e.g. from the high-power self condition we subtracted the high-power control condition). Next, we entered these contrasts to two 2nd-level multiple regressions with LSAS scores as a covariate. The resulting activation maps were thresholded with a voxel-level threshold of *p* < 0.001 (Eklund, Nichols, & Knutsson, 2016; Woo, Krishnan, & Wager, 2014) and cluster-level correction at pFWE < 0.05. For basic validation of the SRET, we computed a contrast estimating the effect of self *v.* control (i.e. summation of self *v.* control blocks) and subjected these contrasts to a group-level one-sample *t* test. Group-level effects of the tasks conditions were inspected as well in exploratory analyses.

#### Region of Interest (ROI) Representational Similarity Analysis (RSA)

In order to better interpret the underlying affective processes of self-evaluation that were mediated by subcortical regions as a function of SA, we conducted an exploratory RSA between the SRET and each of the auxiliary tasks. We assumed that higher between-task similarity would indicate to some degree that participants engaged reward- or threat-related neural representations in a given ROI when self-evaluating (Chavez et al., 2017). In the SRET, we focused our analysis on the high-power self > high-power control contrast, due to association of LSAS with both negative self-evaluation and altered brain activity in response to this condition (see Results). We extracted the following contrasts from the auxiliary tasks (see online Supplementary Methods for details on GLM construction in these tasks): from the reward task, we extracted one contrast reflecting reward responsivity (winning > losing money), and another contrast capturing incentive salience (anticipation > implicit baseline). From the threat task, we extracted one contrast representing sensitivity to social threat cues (fearful + angry faces > shapes; hereinafter termed ‘negative faces’). We focused our analysis on a right VS and left VMPFC ROIs [regions no. 220 and 47 in the Brainnetome atlas; (Fan et al., 2016)], since LSAS correlated with response to high-power in these regions (see Results). We then correlated the similarity values with LSAS scores, and controlled for multiple comparisons by applying a pFDR ⩽ 0.05 threshold on the emerging results for each ROI.

Since RSA is a correlation-based measure and thus sensitive to the number of voxels, we wanted to assure that a similar amount of voxels was available for all participants. Thus, we re-created the ROIs based on their overlap with the grouped-average brain mask produced during the 2nd-level analysis. The right VS contained 162 voxels and the left VMPFC contained 130 voxels. This analysis was carried with the RSA toolbox (Nili et al., 2014), and its computation pipeline is schematically depicted in Fig. 4a.

## Results

### Reduced endorsement of power v. affiliation traits in SA

**Fig. 2.**
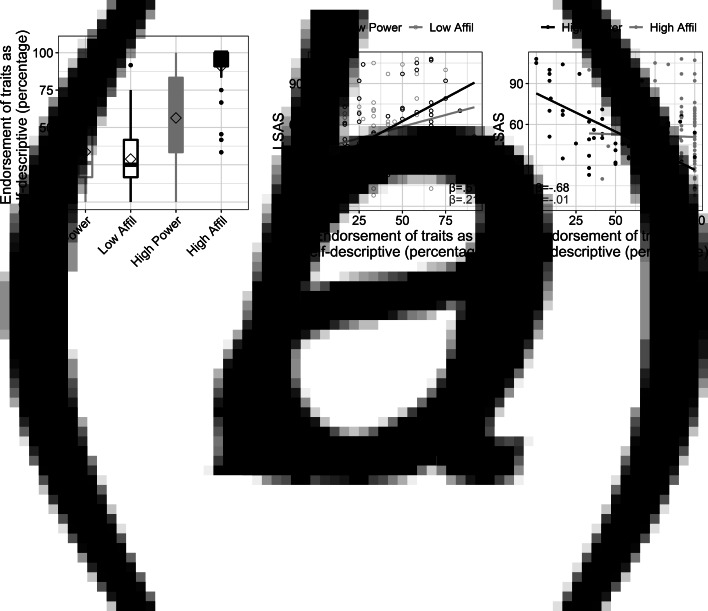
Endorsement of traits according to their social domain and intensity, and its interaction with social anxiety. (a). Boxplots depicting the percentage of endorsing traits as self-descriptive (i.e. responding ‘yes’ to the question ‘does the trait describe me?’) in each of the four experimental conditions. The power v. affiliation (abbreviated as affil) social domains are colored in black and gray, respectively. High v. low intensity is denoted by color fill or empty fill, respectively. The horizontal line in each boxplot marks the median, and upper and lower bounds of the box denote the 75th (Q3) and 25th (Q1) percentiles, respectively. The line stretches between the minimum (Q1 − 1.5 × interquartile range) and maximum (Q3 + 1.5 × interquartile range) of the data. The diamond shape indicates the mean. (b). Exploration of the 3-way interaction of social anxiety level, as measured by the Liebowitz Social Anxiety Scale (LSAS), with intensity and social domain of traits. The interaction was explored by computing and comparing between simple slopes that quantified the association of LSAS (*y*-axis) with endorsing traits within each condition. The simple slopes for LSAS (*β*-value) within each of the traits' conditions are overlaid on each scatterplot and are colored according to the social domain.

We first examined whether the domain and intensity of traits in the SRET, as well as their interaction with LSAS scores, predicted the percentages of endorsing traits as self-descriptive. This linear regression was significant (*R*^2^ = 0.45, Adjusted *R*^2^ = 0.43, *F*_(7,288)_ = 33.39, *p* < 0.001). At the group-level, there were significant effects for social domain and its interaction with intensity on endorsement percentages, when LSAS was not included in the model (i.e. via repeated-measures ANOVA). These effects were mostly driven by the high endorsement of high-affiliation traits (Fig. 2a; see online Supplementary Tables S4–S6 for regression and repeated-measures ANOVA results, and online Supplementary Table S3 for descriptive statistics of the SRET).

In accordance with our hypothesis, SA-severity associated negatively with self-evaluation in terms of power [simple slope: *t*(288) = −7.98, *p* < 0.001] rather than affiliation [simple slope: *t*(288) = −0.13, *p* = 0.9; two-way interaction between LSAS and social domain: *β* = −0.83, *t* = −5.55, *p* < 0.001]. The analysis also revealed a significant three-way interaction between LSAS, domain and intensity of traits (*β* = 0.37, *t* = 2.13, *p* = 0.03; Fig. 2b). Post-hoc slope tests indicated that SA-severity was associated with negative self-evaluation in terms of power relative to affiliation within each intensity loading, but this effect was particularly robust within high-intensity traits [slope differences test for high-power *v.* high-affiliation: *t*(288) = −5.55, *p* < 0.001; for low-power *v.* low-affiliation: *t*(288) = −2.53, *p* = 0.011; see simple slope coefficients in Fig. 2b]. These results highlight that the association of SA-severity with negative self-evaluation in the power *v.* affiliation domain was more pronounced within the high end of the power and affiliation continua. Note that effects of the SRET conditions on response time in the self conditions, as well on accuracy and response time in the control condition, are presented in the online Supplementary Results and Tables S7–S9.

### fMRI results

#### Association of SA with high-power in VS and VMPFC

Turning to the fMRI data, we first verified that the SRET successfully replicated previous studies by associating self-referential *v.* control conditions with DMN activity. Notably, in the self-evaluation condition subcortical regions of interest (amygdala, VS) more strongly activated than in the control condition (online Supplementary Fig. S4). Group-level effects found for social domain and intensity of traits, as well as for the interaction of these factors, are presented in online Supplementary Table S10.

**Fig. 3.**
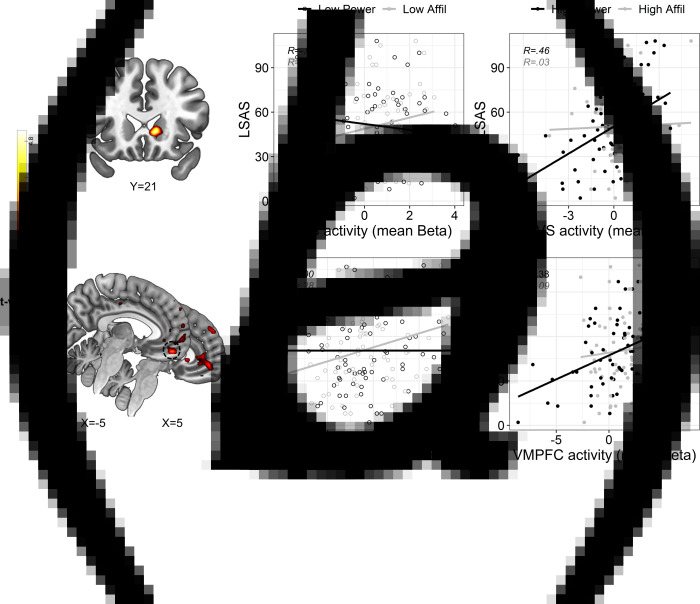
Modulation of brain activity by the interaction between social anxiety, domain and trait intensity. (a). Statistical parametric maps resulting from a whole-brain analysis of the 3-way interaction. Significant interaction effects were revealed in clusters in the right VS (upper panel) and VMPFC (lower panel). Statistical threshold was set at a voxel-level *p* < 0.001 and cluster-level pFWE < 0.05. Approximate location of a peak activation in the VMPFC that was used for post-hoc analysis is circled in a dashed black line in the lower panel. (b). Scatterplots depicting the correlation between SA-severity and brain activity during self-evaluation of low-and high-intensity traits (presented on the left v. right panels, respectively, with empty v. color fill) pertaining to affiliation v. power domains (colored in black v. gray, respectively), in the right VS and VMPFC (upper and lower panel, respectively). The plotted Beta values represent brain activity that was extracted from contrasting each self condition against its matching control condition. Linear trend lines and correlation coefficients (Pearson's R) are presented in each scatterplot and are colored according to their social domain, solely for illustrative purposes. Abbreviations: Right ventral striatum (R.VS); ventromedial prefrontal cortex (VMPFC); affiliation (affil). Brain images are presented in neurological convention (i.e. right is right).

**Fig. 4.**
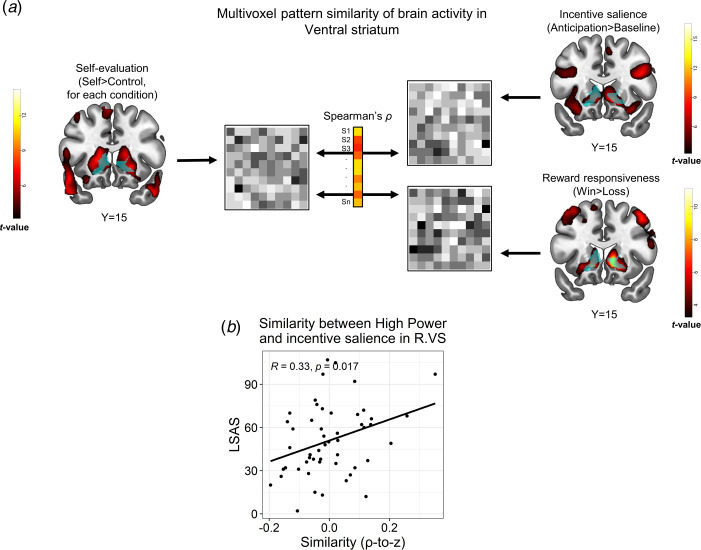
Representational similarity analysis (RSA) between the self-referential encoding task and an auxiliary task probing reward processes. (a). Schema of the between-task RSA pipeline. Multivoxel brain activity patterns in a VS ROI (light blue) were extracted from a relevant contrast in the SRET (self > control for a specific condition; left panel), as well as from contrasts in the reward task that represented incentive salience processing (anticipation > baseline) and reward responsiveness (win > loss). Next, these patterns were correlated for each subject, thus yielding a Spearman's correlation coefficient, which represented the between-task similarity for that subject. We then applied a Fischer *r*-to-*z* transformation on the correlation coefficients and correlated them with LSAS scores across participants. (b). Correlations between LSAS scores and similarity of self-evaluation of high-power with incentive salience in the VS (right). Pearson's correlation coefficient and its corresponding *p* values are overlaid on the scatterplot. Abbreviations: right (R); ventral striatum (VS); Liebowitz social anxiety scale (LSAS). The statistical parametric maps show the group-level effect (i.e. *t* values of a one sample *t* test) obtained for each contrast (note that the map from the SRET shows the self > control contrast across all conditions) and are thresholded at voxel-level *p* < 0.001. All brain images are displayed in neurological convention.

Next, we examined the hypothesis that SA-severity would be associated with altered DMN and subcortical activity specifically during self-referential processing of *power* traits. While the 2-way interaction between LSAS and social domain did not reveal significant effects, we found a significant 3-way interaction between LSAS scores, social domain, and intensity in the VMPFC and right VS – specifically the ventral caudate (Fig. 3a and online Supplementary Table S11). To explore the interaction, we extracted mean activity (beta value) from 4 mm spherical ROIs centered around peak activations in the VMPFC (*x* = −5, *y* = 49, *z* = −19) and right VS (*x* = 11, *y* = 21, *z* = −1) for each self-evaluation condition (*v.* control), and plotted these values against LSAS scores. In partial correspondence with our hypothesis, this post-hoc inspection revealed that SA-severity was associated with enhanced VMPFC activity specifically during the processing of high-power traits; whereas this pattern was not evident for high-affiliation traits. In addition, SA-severity was also positively associated with VS activity during self-evaluation of high-power (our hypothesis regarding this region was two-sided). Moderate or weak associations between SA-severity and brain activity were evident in the remaining conditions (Fig. 3b). Exploratory analyses revealed that higher activity in the above-defined VMPFC and right VS spherical ROIs also associated with less endorsement of high-power traits [VMPFC: *r*(69) = −0.26, *p* = 0.03; right VS: *r*(69) = −0.34, *p* = 0.004]. However, the positive association with LSAS in the VMPFC and VS was only slightly attenuated after controlling for endorsement percentages [VMPFC: *r*(68) = 0.29; right VS: *r*(68) = 0.34; compare with Fig. 4b]. Thus, VMPFC and VS activity in response to high-power was uniquely correlated with SA-severity above and beyond the endorsement of these traits as self-descriptive.

#### Similarity between self-referential encoding of high-power and reward processing in VS associates with SA

We next tested if brain activity during self-referential processing, possibly mediated reward- or threat-related processes in correspondence with SA. We focused this analysis on high-power, due to the association of LSAS with both negative self-evaluation and altered brain activity in response to this condition; and on right VS and left VMPFC clusters wherein LSAS interacted with high-power (Fig. 3). We found that during self-referential encoding of high-power, individuals with higher SA-severity were more likely to activate a multivoxel pattern in the right VS that resembled a pattern evoked by incentive salience in the reward task [*r*(50) = 0.33, *p* = 0.017, pFDR = 0.05, Fig. 4b]. Moreover, in the right VS ROI, LSAS was not related to similarity of high-power to either reward *responsiveness* in the reward task [*r*(50) = −0.12, *p* = 0.39, pFDR > 0.59], nor to threat processing in the negative faces task [r(65) = −0.01, *p* = 0.94, pFDR > 0.94]. Additional control analyses did not reveal similar correlations of LSAS with similarity between self-processing of traits and incentive salience in the remaining SRET conditions (online Supplementary Table S12); nor with endorsing high-power traits [*r*(50) = −0.17, *p* = 0.22]. For the left VMPFC ROI, no significant correlations were found between LSAS scores and similarity of the high-power contrast to any of the contrasts in the auxiliary tasks [incentive salience: *r*(50) = 0.14, *p* = 0.33; reward responsiveness: *r*(50) = −0.11, *p* = 0.44; threat: *r*(65) = −0.07, *p* = 0.58]. Note that RSA analyses conducted at the whole-brain level revealed a significant group-level effect for similarity between self-evaluation (SRET) and incentive salience processing (reward task) within the VS (online Supplementary Fig. S5); and also a correlation between LSAS and similarity of high-power to incentive salience in the VS and VMPFC, albeit at an uncorrected statistical threshold (online Supplementary Fig. S6). These additional findings enhance the reliability of the between-task similarity analysis and corroborate the ROI RSA results.

## Discussion

In this study, we examined the neural and affective processing of self-views in the domains of power and affiliation in SA. As expected, SA-severity was negatively associated with self-evaluation in the power domain, thereby corresponding to indications of impaired self-views in the domain of power *v.* relatively positive affiliation self-evaluation in clinical and subclinical SA (Berger et al., 2017; Gilboa-Schechtman et al., 2017). However, at the neural level, SA-severity associated with altered neural activity only during self-evaluation of high-power traits; specifically, with increased VS and VMPFC activity. Moreover, during self-processing of high-power traits, individuals with higher SA showed brain activity patterns that were more similar to those evoked by incentive salience in the VS.

Our finding that SA-severity was associated with increased activity in the VMPFC and right VS (ventral caudate) specifically during self-evaluation of high-power, both replicates and extends findings from previous examinations of negative self-evaluation in SA. Enhanced MPFC is recurrently evident during self-referential processing in clinically diagnosed SA (see Yoon et al., 2019 for a review). In specific, the foci of MPFC activations found here overlap with areas in the VMPFC and pregenual anterior cingulate cortex, which have been recurrently associated with processing valuable self-relevant information (Bartra et al., 2013; D'Argembeau et al., 2012; Lieberman, Straccia, Meyer, Du, & Tan, 2019; Qin & Northoff, 2011). Similar areas were previously found hyper-activated in SA in response to both negative and positive self-related information such as self-beliefs and social feedbacks (Goldin et al., 2009a, 2009b; Heitmann et al., 2014; Peterburs et al., 2016). Thus, enhanced VMPFC activity may reflect enhanced sensitivity to information that is valuable to the self in SA, regardless of its valence, and could possibly also indicate excessive scrutiny of one's self-views with regards to that information.

Findings on aberrant VS activity in SA are accumulating as well. Neuroimaging studies of SA found enhanced VS response to social and non-social threats (Boehme et al., 2014; Brühl et al., 2011; Klumpp et al., 2012), as well as VS hypoactivation during anticipation and consumption of social rewards (Becker et al., 2017; Richey et al., 2017, 2014; Schultz et al., 2019). Here we extend this line of findings by revealing altered VS activity in SA during self-evaluation of specific social content, and by suggesting a specific affective process that the VS may mediate during this context. The VS, and specifically the ventral caudate, track the amount of effort people put into gaining rewards (i.e. their degree of ‘wanting’ rewards) (Croxson, Walton, O'Reilly, Behrens, & Rushworth, 2009; Miller, Shankar, Knutson, & McClure, 2014). Furthermore, both the VS and the VMPFC, which were co-activated here, are central components of a brain system that signals valuable self-relevant information and potentially rewarding outcomes (Bartra et al., 2013; D'Argembeau et al., 2012; Liu et al., 2011). These functions align with the assignment of enhanced motivational significance to power-related content in SA (Kashdan et al., 2008; Roberts et al., 2011). Moreover, high-power traits may constitute a type of ‘ideal self’ for people with high SA; namely a desirable trait that not only holds high incentive value, but is also uncharacteristic of oneself (Higgins, 1987; Markus & Nurius, 1986). Respectively, people with high SA may experience a large discrepancy between their perception of their own power and their actual social performance in their everyday lives (Kashdan et al., 2008; Roberts et al., 2011) – a deviance that may also be mediated via the VS and VMPFC (Will, Rutledge, Moutoussis, & Dolan, 2017; Yoon et al., 2018).

Here, the association of VS activity with signaling the high value of high-power traits on the one hand and their mismatch with regards to one's own self-view in SA on the other, were corroborated by two additional analyses. First, stronger VS and VMPFC responses to high-power were also correlated with lower endorsement of these traits. Second, brain activity patterns in the right VS during processing of high-power traits resembled those evoked by incentive salience in the reward task (i.e. a reward anticipation phase), as a function of SA-severity. This may suggest that this type of reward-related processing was activated to some extent in the VS during self-evaluation of high-power as a function of higher SA. However, note that while high-power traits were rated as more positive than low-power traits (see online Supplementary Methods), we did not directly assess participants' subjective evaluations of these traits as desirable or important. Furthermore, it is important to note that previous studies showed that the VS-VMPFC circuit is associated with trait self-esteem (Chavez & Heatherton, 2014, 2017). To exclude the possibility that the correlations we found between LSAS and VS-VMPFC activity are better explained by self-esteem, we show that all results remain significant also while controlling for scores on the self-esteem questionnaire (see online Supplementary Results and Tables S13 and S14).

This study also demonstrates how the methodology of between-task similarity can enhance the interpretability of subcortical activity evident during complex cognitive tasks such as self-evaluation. By administering auxiliary fMRI tasks that probe basic affective processes, we were able to demonstrate that activation of similar multi-voxel brain activation patterns across the SRET and the reward task in the VS scaled with SA-severity – thereby suggesting the embedment of a core affective process (i.e. striatal signaling of incentive salience) in self-evaluation. Our approach was inspired by recent studies, which by implementing between-task similarity demonstrated that positive affect and additional mental functions (e.g. language, scene construction) operate during tasks of internal mentation (Axelrod et al., 2017; Chavez et al., 2017). A more a recent study showed that participants who activated multivoxel brain activity patterns while viewing neutral pictures that were more similar to those activated during exposure to negative pictures, reported on more positive affect and less negative affect in their daily lives (Puccetti et al., 2021). Together with the results presented here, these findings demonstrate how individual differences in the degree of similarity between a mental state and a certain affective context, may help provide a more precise delineation of the affective neural processes that govern psychopathology symptoms.

Findings from this study may have clinical implications. SA treatment could benefit from identifying problematic self-views underlying this condition. Clinicians may focus on the way patients relate to high-power content, as well as on resolving conflictual motivations associated with such self-views. At the neural level, hitherto studies examining neural changes related to therapeutic effects in SA have largely focused on attenuating amygdala reactivity and on up-regulating fronto-parietal cortical networks that support explicit emotional regulation (Dixon et al., 2020; Goldin et al., 2013; Klumpp et al., 2017; Klumpp & Fitzgerald, 2018; Phan et al., 2013). In contrast, our findings suggest that downregulating VMPFC and VS activity, possibly mediating the excessive engagement of implicit value-related self processes (e.g. I am not good enough), may serve as an additional clinical target in SA – at least in the context of processing self-related content. Gaining a more nuanced understanding of which neural domain should be targeted under different contexts in order to effectively treat psychopathology symptoms, can also inform novel neuro-modulation based interventions such as process-based neurofeedback (Lubianiker et al., 2019).

### Limitations

A number of limitations of this study should be taken into account. The valence of traits in the SRET differed between conditions, as low-intensity conditions consisted mostly of negatively valenced traits whereas high-intensity traits were positively valenced (and were especially positive in the high-affiliation conditions; see online Supplementary Results). Thus, although online Supplementary analyses suggested that traits were categorized and endorsed in a manner that reflected sensitivity to their social domain (see online Supplementary Results and Tables S1 and S2), we cannot determine the extent to which the results were driven by the valence of traits rather than their social domain. Relatedly, the high and uniform endorsement rates of high-affiliation traits suggest that the SRET was less sensitive to measurements of individual differences with respect to this domain. Future studies of power and affiliation could gain better control over these issues by equating valence across categories. In addition, the association of low-intensity traits with their designated category was less robust compared to the high-intensity traits, and it is possible that they could be classified more accurately using a different categorization framework. Future studies could yield more precise profiling of traits by taking into account multiple social domain axes on which they may be represented. Moreover, although clinical levels of SA that meet with the criteria for diagnosing SAD were evident in our sample, it remains an open question whether findings from this study would arise in a SAD patients; and perhaps if such patients would exhibit aberrant activation in regions of interest that were absent here, such as the amygdala.

In conclusion, consistent with cognitive-evolutionary accounts of SA (Gilbert, 2001), our study emphasizes the centrality of power-related self-views in this condition. We show that excessive VS and VMPFC activation accompany self-referential encoding of high-power traits as a function of SA-severity, and suggest that patterns of activations may indicate enhanced significance of such traits for one's ideal self-view. Our findings thus ratify theoretical perspectives that entangle aberrant self-evaluation in psychopathology in general, and in SA in particular, with affective processes (Frewen et al., 2020; Higgins, 1987; Markus & Nurius, 1986).

## Supplementary material

For supplementary material accompanying this paper visit https://doi.org/10.1017/S0033291722001453. click here to view supplementary material
