# Supplementary material for: Self-evaluation of social-rank in socially anxious individuals associates with enhanced striatal reward function
Source: Psychol Med. 2022 Jun 14;53(10):4569–79. doi: 10.1017/S0033291722001453 (PMC10388315; doi:10.1017/S0033291722001453)
Supplement: Supplementary file 1 [file S0033291722001453sup001.docx]

**Supplementary Information**

Supplementary Methods

Online screening procedure of participants

Figure S1. Distributions of LSAS scores

Stimuli validation experiment

Table S1. The selected power and affiliation stimuli

Table S2. Validation of power and affiliation stimuli – categorical Principal Components Analysis (PCA)

Auxiliary fMRI tasks for characterizing basic evaluative processes

Monetary reward task

Figure S2. Design of the monetary reward task

Negative faces task

Figure S3. Design of the negative faces task

Statistical analysis of fMRI data from the auxiliary tasks

Removal of one block of the SRET from analyses

Imaging data acquisition

Functional Magnetic Resonance Imaging (fMRI) preprocessing

Supplementary Results

Supplemental behavioral results

Table S3. Descriptive statistics of the SRET

Endorsement of traits in the SRET

Table S4. Regression results for predicting endorsement percentages in the SRET

Table S5. Results from a repeated-measures ANOVA on endorsement percentages in the SRET

Table S6. Differences in endorsement percentages in the SRET

Analysis of response time in the self condition

Table S7. Results from a repeated-measures ANOVA on response time during self conditions in the SRET

Table S8. Differences in response time in the SRET during self conditions

Analysis of accuracy and response time in the control condition

Table S9. Results from a repeated-measures ANOVA on accuracy and response time during control conditions in the SRET

Supplemental fMRI results

Brain activation

Figure S4. Self-referential processing of traits in the SRET

Table S10. Effects of social domain and intensity on brain activation in the SRET

Table S11. Modulation of brain activation by the interaction of social anxiety with traits’ social domain and intensity

Representational Similarity Analysis (RSA)

Table S12. Association of between-task similarity with social anxiety in all conditions

Figure S5. Similarity between self-evaluation and incentive salience

Figure S6. Correlation of social anxiety with similarity between self-evaluation of high-power and incentive salience

Replication of results while controlling for self-esteem

Table S13. Regression results for predicting endorsement percentages in the SRET while controlling for self-esteem

Table S14. Modulation of brain activation by the interaction of social anxiety with traits’ social domain and intensity while controlling for self-esteem

Supplementary Methods

Online screening procedure of participants

Potential participants (n=845 who met the inclusion criteria which are detailed below) were recruited via advertisement on social media, wherein the experiment’s subject was defined as “an examination of the neural mechanisms involved in processing emotional and cognitive aspects of self-perception”. The experiment’s advertisement included a link directing potential participants to complete a short screening form in the *qualtrics* software. The goal of the screening procedure was to assure the recruitment of participants with a wide range of social anxiety symptoms, from about the same age range (21-35), and who also met the criteria for MRI scanning and spoke Hebrew at a mother-tongue level. The screening procedure initiated by electronically signing an informed consent according to the Tel-Aviv University institutional review board (IRB). Afterwards, the potential participants completed two self-report questionnaires addressing social anxiety and self-esteem, whose presentation order was randomized: the Liebowitz Social Anxiety Scale-Self-Report (LSAS-SR) (Fresco et al., 2001; Liebowitz, 1987), and the Rosenberg Self-esteem scale (RSE) (Rosenberg, 1965). We included two questionnaires, as we did not want to expose the study’s focus on social anxiety. Next, participants provided demographic and medical details. Participants were asked to report and elaborate on the existence of any past or present diagnosis of any kind of illness they have, including neurological or psychiatric conditions. We also asked if they take any medications, and specifically asked if they had a diagnosis of ADHD and/or autism/Asperger’s disorder/ pervasive developmental disorder (PDD). Participants were also asked to indicate whether they are right- or left-handed. Lastly, participants filled a standard MRI safety questionnaire. In the screening form, we informed the potential participants that if they will eventually participate in the study, they will be paid for the ~15 min they invested in completing the screening form. All recruited participants were right-handed, and had at least 12 years of education, no reported history of psychiatric or neurological disorders (including ADHD), no current use of psychoactive drugs and spoke Hebrew at mother-tongue level.

The size of the screening sample was not pre-determined. Moreover, we did not start recruitment after all potential participants completed the screening; rather, we initiated the recruitment and continued screening simultaneously. We did this in order to minimize the interval between the screening and the experiment, so that social anxiety levels will remain similar between measurements. Thus, the screening sample size was largely affected by the accumulation of potential participants across time, and it includes many participants that we did not contact. Throughout the screening process, we assured the coverage of a wide range of social anxiety symptoms by relying on established cut-off scores for detecting social anxiety with the LSAS questionnaire. Specifically, cut-off scores of 30 (and also 35) and 50 are typically used to distinguish low- versus high- levels of social anxiety that are typically evident in social anxiety disorder (Lazarov et al., 2021; Mennin et al., 2002; Rytwinski et al., 2009; von Glischinski et al., 2018). A LSAS score of 60 or more is considered to indicate generalized social anxiety disorder (Mennin et al., 2002). We considered these cut-off points during screening by assuring that a sufficient number of participants was available for each range. Thus, 20 participants had LSAS scores of up to 35 (14 of them had less than 30); 18 participants had mid-range scores (36-49); and 36 participants had a LSAS score of 50 or more (30 of these participants had 60 or more). The distribution of LSAS scores is presented in Fig. S1 below.

*
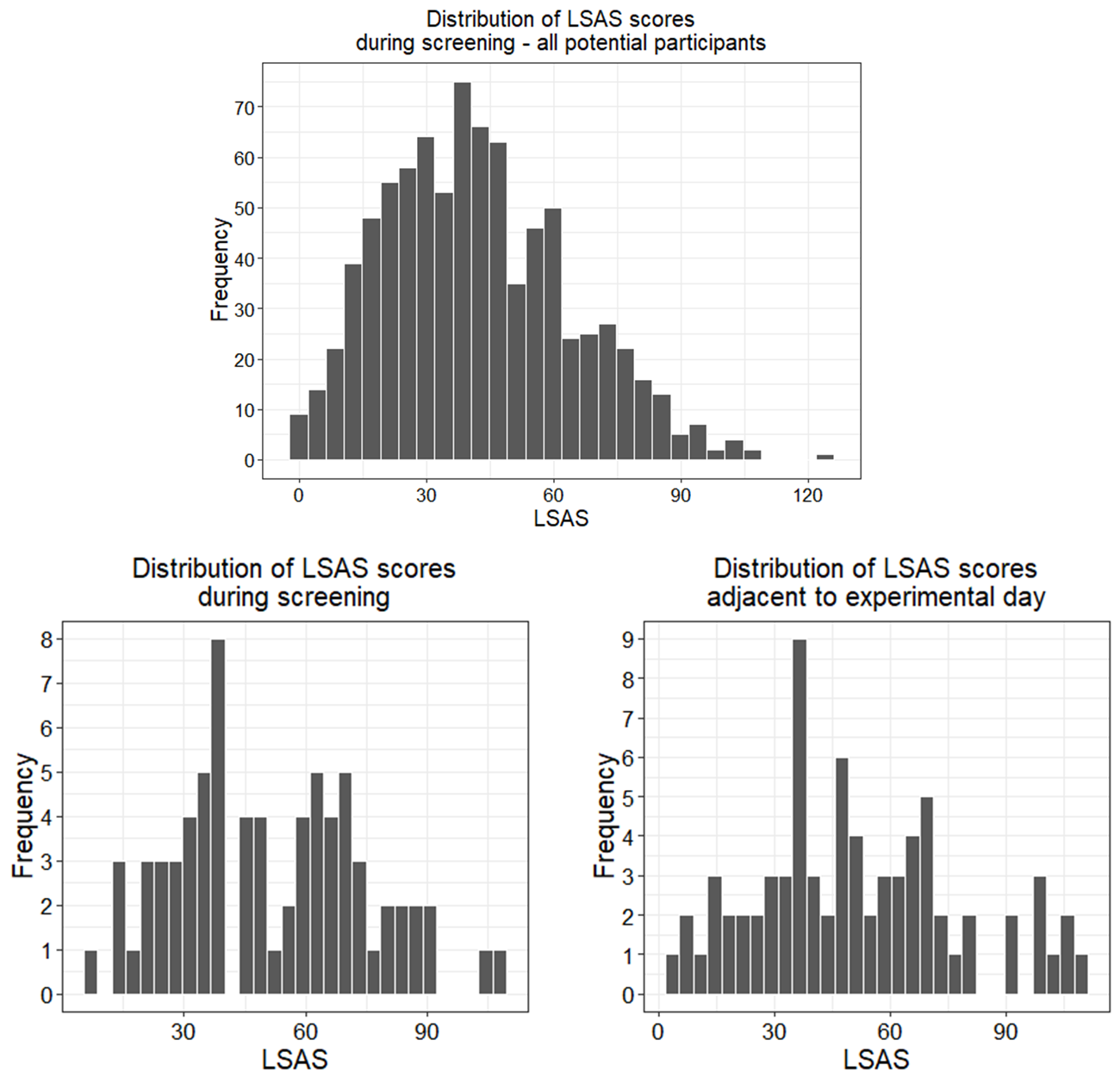
*

**Figure S1. Distributions of LSAS scores.** Histograms depicting the distribution of participants’ scores on the Liebowitz Social Anxiety Scale (LSAS) (Liebowitz, 1987). Higher LSAS scores indicate more severe symptoms of social anxiety. Upper panel: distribution of LSAS score obtained for all potential participants who met inclusion criteria during screening (n=845; *M*±*SD*: 41.86 ± 21.86). Lower panel: distribution of LSAS scores for participants who took part in the experiment. The questionnaire was completed twice: during screening (left lower panel; *M*±*SD*: 51.35 ± 23.07) and 1-2 days before the experimental day (right lower panel; *M*±*SD*: 51.15 ± 26.12). The correlation between time-points was high: r(72)=.86, p<.001.

Stimuli validation experiment

In order to select social trait adjectives that relate to social power and affiliation, as well as to characterize the valence of traits, we performed the following procedure. First, we generated 4 lists of 25 traits, such that each list contained traits that potentially corresponded with two social categories and two intensities, namely high-power (i.e. dominant), low-power (i.e. submissive), high-affiliation (i.e. friendly) and low-affiliation (i.e. hostile). Following (Gilboa-Schechtman et al., 2017), the stimuli were selected based on a review of English studies on relevant personality traits (Anderson, 1968; Haines & Kray, 2005; Keith Campbell, Bosson, Goheen, Lakey, & Kernis, 2007; Raskin & Terry, 1988; Wiggins, 1979; Wiggins & Broughton, 1991; Wojciszke & Sobiczewska, 2013), as well as on one study that specifically used Hebrew words of power and affiliation (Gilboa-Schechtman et al., 2017). Traits from the English studies were translated into Hebrew.

Next, we conducted an online experiment on undergraduate psychology students from Tel-Aviv University (n=43), who received course credit for their participation. The experiment was conducted using the *qualtrics* software. During the experiment, participants were first exposed to a short theoretical explanation about the concepts of power and affiliation. Then, participants were instructed to classify the 100 traits according to their category and intensity – either high-power, low-power, high-affiliation, low-affiliation or “other”, in case that the trait did not match any of the categories. This was executed by dragging each trait from the list and dropping it in a panel that corresponded with the label the participant found most appropriate for that trait. Next, participants classified the same set of traits according to their valence – either positive, neutral or negative. We included this additional classification task since we expected the highly valued and possibly socially desirable traits to have a positive valence, and the low valued and possibly socially undesirable traits to have a negative valence. For each trait, this experiment yielded percentages describing its classification in terms of social category by intensity (first classification task), as well as in terms of valence. Then, from each of the 4 lists we selected 16 traits with the highest categorization percentage in accordance with our designated classification of social category and intensity (i.e. the first classification task). All traits were categorized by at least 60% of the participants as matching with our pre-defined classification (Gilead, Katzir, Eyal, & Liberman, 2016), except for one trait in the low affiliation list that reached 58.14%.

Using a series of Chi-squared tests, we assessed whether participants classified the chosen stimuli according to the pre-defined social categories and intensities. This analysis confirmed that each stimuli set associated with its corresponding *a priori* defined categorization more often than with the 3 other categories and the “other” label (high-power: *χ²*(4)=139.42, p<.001; low-power: *χ²*(4)=102.32, p<.001; high-affiliation: *χ²*(4)=139.84, p<.001; low-affiliation: *χ²*(4)=76.40, p<.001). Next, we examined whether high-intensity traits in both categories were also more often associated with positive valence rather than with negative or neutral valence; and if low-intensity traits were more often associated with negative valence rather than with positive or neutral valence. Chi-squared tests confirmed that the stimuli sets associated with these affective qualities (high-power: *χ²*(2)=51.60, p<.001; low-power: *χ²*(2)=38.16, p<.001; high-affiliation: *χ²*(2)=81.03, p<.001; low-affiliation: *χ²*(2)=37.56, p<.001). The 64 traits used in this study, as well as their percentage of compatibility with the pre-defined social categories and hypothesized valence, are detailed in Table S1.

Lastly, we examined whether the selected traits clustered into two distinct factors according to their social categories (i.e. power vs. affiliation), based on the actual self-evaluations made by participants during the SRET. We first performed a principal component analysis (PCA) on participants’ mean percentages of endorsing traits as self-descriptive (i.e. 4 mean endorsement percentages per participant, 1 per condition). The analysis revealed two components: a power component (Eigenvalue = 1.81, explained variance = 45.14%), on which the endorsement of high-power loaded positively (.89) and that of low-power loaded negatively (-.83); and an affiliation component (Eigenvalue = 1.19 explained variance = 29.77%), on which the endorsement of high-affiliation loaded positively (.89) and that of low-affiliation loaded negatively (-.75). Next, we examined if each trait clustered onto its a-priori defined social category. To this end, we conducted a categorical PCA on all traits from the SRET using SPSS Version 27 (IBM). Two components were extracted from the categorical PCA (Table S2): the first component (Cronbach’s Alpha = .88, Eigenvalue = 7.58, explained variance = 15.8%) was loaded highly by nearly almost all of the traits (23/24) that were categorized as related to power in the independent validation experiment, but also by 6 affiliation-related traits (5 low-affiliation, 1 high-affiliation). The second component (Cronbach’s Alpha = .87, Eigenvalue = 6.85, explained variance = 14.27%) was loaded highly by most of the traits (18/24) that were categorized as related to affiliation, and also by one low-power trait.

| **High-Power** | | | | **Low-Power** | | | |
| --- | --- | --- | --- | --- | --- | --- | --- |
| Trait | |  |  | Trait | |  |  |
| English | Hebrew | Match with  category | Match with positive valence | English | Hebrew | Match with  category | Match with negative valence |
| assertive | אסרטיבי | 95.35% | 88.10% | submissive | כנוע | 90.70% | 95.24% |
| leader | מנהיג | 95.35% | 83.33% | weak | חלש | 88.37% | 88.10% |
| influential | משפיע | 95.35% | 83.33% | exploited | מנוצל | 88.37% | - |
| authoritative | סמכותי | 95.35% | 83.33% | influenced | מושפע | 86.05% | 78.57% |
| powerful | עוצמתי | 95.35% | 85.71% | dragged | נגרר | 86.05% | 97.62% |
| decisive | החלטי | 93.02% | 83.33% | obedient | צייתן | 86.05% | 52.38% |
| strong | חזק | 93.02% | 90.48% | passive | פאסיבי | 83.72% | 71.43% |
| confident | בטוח | 90.70% | 88.10% | inferior | נחות | 81.40% | 85.71% |
| charismatic | כריזמטי | 90.70% | 97.62% | dependent | תלותי | 81.40% | 90.48% |
| leading | מוביל | 90.70% | 88.10% | lenient | ותרן | 79.07% | 71.43% |
| persuasive | משכנע | 90.70% | 80.95% | apologetic | מתנצל | 79.07% | 47.62% |
| determined | נחוש | 90.70% | 95.24% | coward | פחדן | 79.07% | 88.10% |
| independent | עצמאי | 90.70% | 92.86% | hesitant | הססן | 76.74% | 66.67% |
| prominent | בולט | 88.37% | 64.29% | loser | לוזר | 74.42% | 97.62% |
| capable | מסוגל | 88.37% | 97.62% | vulnerable | פגיע | 72.09% | 59.52% |
| dominant | שולט | 88.37% | 52.38% | gullible | פתי | 72.09% | 73.81% |
|  |  |  |  |  |  |  |  |
| **High-Affiliation** | | | | **Low-Affiliation** | | | |
| Trait | |  |  | Trait | |  |  |
| English | Hebrew | Match with  category | Match with positive valence | English | Hebrew | Match with  category | Match with negative valence |
| empathetic | אמפתי | 97.67% | 100.00% | distant | מרוחק | 93.02% | 80.95% |
| kind | אדיב | 95.35% | 97.62% | opaque | אטום | 83.72% | 95.24% |
| cordial | לבבי | 95.35% | 97.62% | mean | מרושע | 81.40% | 95.24% |
| pleasant | נעים | 95.35% | 100.00% | cold | קר | 81.40% | 88.10% |
| caring | אכפתי | 93.02% | 100.00% | detached | מנותק | 79.07% | 83.33% |
| friendly | חברותי | 93.02% | 100.00% | loner | מתבודד | 76.74% | 59.52% |
| considerate | מתחשב | 93.02% | 100.00% | indifferent | אדיש | 72.09% | 57.14% |
| generous | נדיב | 93.02% | 97.62% | insulting | מעליב | 72.09% | 95.24% |
| helpful | עוזר | 93.02% | 100.00% | nasty | נבזי | 72.09% | 95.24% |
| containing | מכיל | 90.70% | 97.62% | shut | סגור | 72.09% | 69.05% |
| patient | סבלני | 90.70% | 97.62% | offensive | פוגעני | 69.77% | 95.24% |
| sympathetic | סימפטי | 90.70% | 100.00% | cynical | ציני | 69.77% | 54.76% |
| attentive | קשוב | 90.70% | 100.00% | sarcastic | עוקצני | 65.12% | 85.71% |
| supportive | תומך | 88.37% | 100.00% | strange | מוזר | 62.79% | 59.52% |
| fun | כיפי | 86.05% | 100.00% | egotistical | אגואיסט | 60.47% | 92.86% |
| open | פתוח | 86.05% | 92.86% | discreet | חשאי | 58.14% | 21.43% |

**Table S1. The selected power and affiliation stimuli.** The Hebrew words that consisted each condition, as well as their possible translation to English, are presented. The percentage of participants that classified each trait as matching with its designated category are presented (“Match with category”), and each list is sorted according to these values (from largest to smallest). The percentage of participants that classified each trait as matching with positive valence (for high-intensity traits) and negative valence (for low-intensity traits) is indicated as well (“Match with positive/negative valence”). Traits that were excluded from analysis due to a technical error are shaded in grey (see below).

|  | **Loadings** | |  |  | **Loadings** | |  |
| --- | --- | --- | --- | --- | --- | --- | --- |
| **Trait** | **Dimension 1 (Power)** | **Dimension 2 (Affiliation)** | **Condition** | **Trait** | **Dimension 1 (Power)** | **Dimension 2 (Affiliation)** | **Condition** |
| capable | -0.596 | 0.171 | hp | insulting | 0.193 | -0.645 | la |
| assertive | -0.590 | -0.362 | hp | nasty | 0.051 | -0.630 | la |
| persuasive | -0.543 | -0.280 | hp | offensive | -0.137 | -0.534 | la |
| powerful | -0.542 | 0.029 | hp | sarcastic | -0.076 | -0.438 | la |
| strong | -0.542 | 0.009 | hp | mean | -0.028 | -0.436 | la |
| authoritative | -0.506 | -0.050 | hp | indifferent | 0.321 | -0.413 | la |
| influential | -0.501 | -0.167 | hp | cynical | 0.159 | -0.372 | la |
| determined | -0.499 | 0.122 | hp | obedient | 0.277 | 0.327 | lp |
| fun | -0.496 | 0.016 | ha | cordial | -0.278 | 0.394 | ha |
| leader | -0.471 | -0.334 | hp | kind | -0.034 | 0.463 | ha |
| decisive | -0.471 | -0.279 | hp | containing | -0.069 | 0.482 | ha |
| prominent | -0.464 | -0.370 | hp | friendly | -0.101 | 0.491 | ha |
| dominant | -0.372 | -0.334 | hp | patient | 0.016 | 0.514 | ha |
| submissive | 0.372 | 0.156 | lp | generous | -0.088 | 0.566 | ha |
| shut | 0.386 | 0.002 | la | caring | -0.108 | 0.574 | ha |
| exploited | 0.401 | 0.138 | lp | considerate | -0.083 | 0.590 | ha |
| hesitant | 0.410 | 0.222 | lp | pleasant | -0.147 | 0.666 | ha |
| vulnerable | 0.410 | 0.323 | lp | attentive | -0.116 | 0.690 | ha |
| loser | 0.433 | -0.416 | lp | empathetic | -0.202 | 0.733 | ha |
| dragged | 0.453 | -0.241 | lp |  |  |  |  |
| coward | 0.486 | 0.240 | lp |  |  |  |  |
| inferior | 0.489 | -0.134 | lp |  |  |  |  |
| dependent | 0.512 | -0.216 | lp |  |  |  |  |
| strange | 0.512 | 0.074 | la |  |  |  |  |
| distant | 0.525 | -0.140 | la |  |  |  |  |
| influenced | 0.526 | 0.197 | lp |  |  |  |  |
| lenient | 0.531 | 0.001 | lp |  |  |  |  |
| loner | 0.549 | 0.084 | la |  |  |  |  |
| detached | 0.646 | -0.225 | la |  |  |  |  |

**Table S2. Validation of power and affiliation stimuli – categorical Principal Components Analysis (PCA).** The table presents loadings for all analyzed traits in the SRET onto two dimensions, as revealed by a categorical PCA. Traits that loaded more highly on the power-/affiliation-related dimensions are displayed on the left/right side of the table, respectively, and are sorted in an ascending manner with regards to the relevant dimension. Traits that were categorized as related to power/affiliation in the independent validation experiment are colored in red/green, respectively. The condition of each trait is indicated in the last column as follows: high-power (hp), low-power (lp), high-affiliation (ha), low-affiliation (la).

Auxiliary fMRI tasks for characterizing basic evaluative processes

Monetary reward task

In order to probe neural activation in reward-related areas of the brain – especially in the VS – we utilized a monetary gambling task (Fig. S2) that was identical to that used by (Carlson, Foti, Mujica-Parodi, Harmon-Jones, & Hajcak, 2011). This task is known to elicit robust activation in the VS. Each trial started with a white fixation cue presented in the center of a black screen (500 ms). Then, two identical doors were presented side-by-side for 4,000 ms. Participants were instructed before the task that behind one of the doors was a monetary prize [+5 New Israeli Shekel (NIS), equivalent to ~$1.4] whereas behind the other door there was a loss (−2.5 NIS). Participants were told that if they did not make their choice while the doors were presented, the computer would select a door randomly. Participants used an MRI-compatible response box to choose one of the doors. Then, after another fixation cue (500 ms), a feedback screen was displayed (1,000 ms) wherein a green arrow indicated a correct guess leading to a monetary prize, while a red arrow indicated monetary loss. Finally, a blank black screen jittered inter-trial interval occurred between each trial (1,500–14,000 ms, *M*=4,000 ms). The task consisted of 60 trials with 30 predetermined wins and 30 losses presented in pseudorandom order and divided equally into two functional runs. That is, unknown to participants, their choice did not influence whether a trial was a win or loss. Prior to the task, participants were instructed about its stages and were informed that the size of the monetary prize will eventually be determined by summing the monetary gains and losses from 12 (out of 60) randomly chosen trials, so that they could earn up to 60 NIS. The experiment was programmed and presented using E-prime software (Psychology Software Tools, Pittsburg, PA). Total task duration was 585 s.


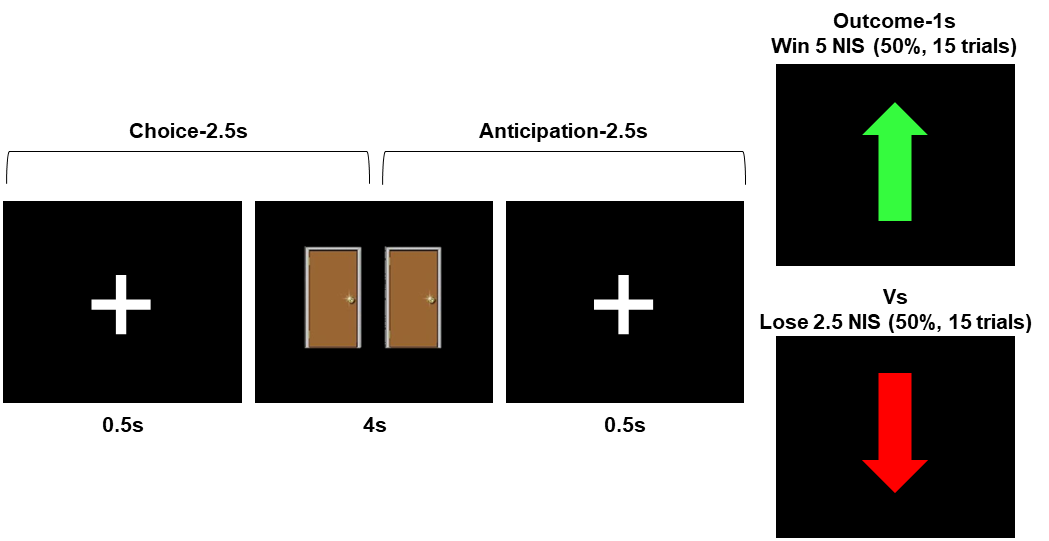


**Figure S2. Design of the monetary reward task.** A schematic illustration of the task design. In each trial, the participant chose between two doors. The choice phase was followed by a short anticipatory phase, and then appearance of an upward pointing green arrow or a downward pointing red arrow indicated either gain or loss of money, respectively.

Negative faces task

Stimulation of threat-related activation in the amygdala was addressed by utilizing an extensively used face-matching task, termed here the “negative faces task”, that is known to elicit robust and replicable amygdala activity (Fisher et al., 2006; Hariri, Tessitore, Mattay, Fera, & Weinberger, 2002; Scult, Knodt, Radtke, Brigidi, & Hariri, 2019; Zhou et al., 2008). The paradigm (Fig. S3) consists of 4 blocks of a perceptual face-matching task interleaved with 5 blocks of a sensorimotor control task. Participants were instructed to select one of two faces/shapes (located at the bottom right or bottom left of the screen) that matched the target face/shape (located at the top of the screen), as accurately and as quickly as possible. Each faces’ block consisted of 6 repetitions of one of four emotional facial expressions - angry, fearful, surprised and neutral. Each sensorimotor control block consisted of 6 different shape trios (consisting of circles and vertical and horizontal ellipses). All blocks were preceded by a brief instruction (“Match Faces” or “Match Shapes”) that lasted 2 s. In the faces’ blocks, each of the 6 face trios was presented for 4 s with a variable inter-stimulus interval (ISI) of 2–6 s (*M*=4 s) for a total block length of 48 s. A variable ISI was used to minimize expectancy effects and resulting habituation, and to maximize amygdala reactivity throughout the paradigm. In the control blocks, each of the 6 shape trios was presented for 4 s with a fixed ISI of 2 s for a total block length of 36 s. Total task time was 390 s. The order of the emotional faces’ blocks was counterbalanced between subjects, by using four different versions consisting of different block orders.


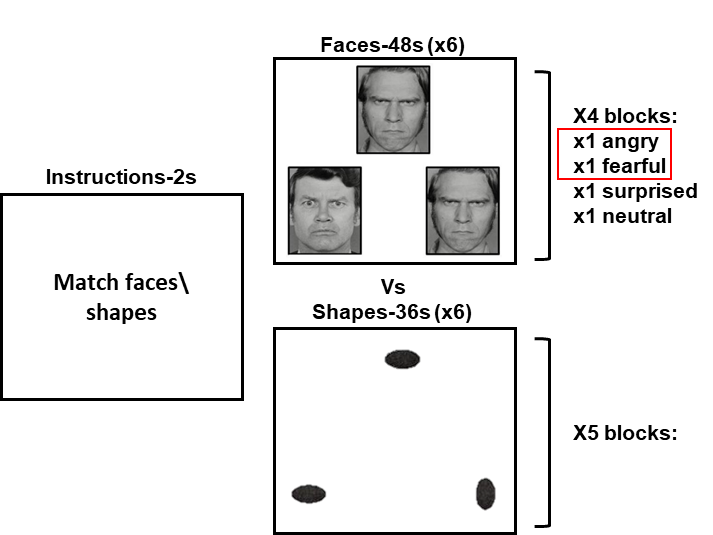


**Figure S3. Design of the negative faces task.** The task consisted of 4 blocks of emotional faces, which alternated with 5 sensorimotor control blocks that were performed on shapes. Note that in the analysis we focused on stimuli that clearly associate with negative valence – namely angry and fearful faces.

Statistical analysis of fMRI data from the auxiliary tasks

Statistical analysis of the fMRI data was conducted with Statistical Parametric Mapping software (SPM12; <http://www.fil.ion.ucl.ac.uk/spm>). We implemented a General Linear Model (GLM) in order to estimate neural responses to the experimental conditions.

In the 1^st^-level model of the monetary reward task, we created predictor variables for the four phases of the task as follows: (1) choice (2.5 s), (2) anticipation (2.5 s), win (i.e. reward; 1 s) and loss (i.e. punishment; 1 s). The “win” and “loss” phases were aligned with the onset of the outcome phase. The period prior to outcome onset was divided into two epochs: The first TR (lasting 2,500 ms) following the doors onset was classified as “choice”, and the following TR was coded as “anticipation” (Erdman et al., 2020). We then computed linear contrasts for win>loss, which represented sensitivity to reward versus punishment; and for anticipation>baseline, which captured reward expectancy and possibly engaged incentive salience processing.

In the 1^st^-level model of the negative faces task, we created predictor variables for the task stages as follows: (1) shapes (36 s), (2)-(5) were four separate predictors for each type of the emotional expression, each lasting 48 s, and (6) instructions (2 s). As we were most interested in neural reactivity to negatively valenced stimuli, we focused on the fearful and angry faces, and calculated a linear contrast that compared them against the control condition (i.e. angry+fearful faces>shapes).

For both tasks, confound regressors from the preprocessing step were included as covariates of no interest in the 1^st^-level GLM, and were identical to those use for the SRET (see fMRI preprocessing section above). In addition, all predictors were convolved with a canonical hemodynamic response function, and data were subjected to SPM12 default high-pass filter cutoff (128 s).

Removal of one block of the SRET from analyses

For 40 participants one block of high-power traits was erroneously presented twice under the self condition, and another block of high-power was presented twice under the control condition. These blocks were discarded from both the behavioral and fMRI analyses. Since we wanted to adjust the number of traits between conditions, we removed one block from each of the three remaining conditions as well. We excluded blocks based on the mean percentage of compatibility of the traits within the block with their corresponding condition, as found in the stimuli validation experiment (see Table S1 above), such that blocks with the lowest compatibility were discarded from the analysis.

Imaging data acquisition

All scans were performed using a Siemens 3T Prisma Magnetom VD13 echo speed scanner with a 20-channel head coil. Structural scans included a T1-weighted 3D axial spoiled gradient echo (SPGR) pulse sequence (repetition time/echo time [TR/TE] = 1,860/2.74 ms, flip angle = 8°, voxel size = 1 x 1 x 1 mm, field of view = 256 × 256 mm, slice thickness = 1 mm).

Functional whole-brain scans of all fMRI tasks were performed in an interleaved bottom-to-top order with a T2*-weighted gradient echo planar imaging pulse sequence (TR/TE = 2500/30 ms, flip angle = 82°, voxel size = 2.3 x 2.3 x 3 mm, field of view = 220 × 220 mm, slice thickness = 3 mm, 42 slices per volume). Note that for the first 20 participants who completed the SRET, TE was 35, flip angle was 90° and 38 slices per volume were obtained. These participants were included in the analysis. In addition, for 3 of the latter 20 participants, slice thickness was set at 3.1 mm due to a technical error. These participants were excluded from the fMRI analysis, as their data was not suitable for group-level analysis. The number of volumes acquired for each of the fMRI tasks was as follows: SRET - 2 sessions including 118 volumes each; monetary reward - 2 sessions including 117 volumes each; negative faces (i.e. threat) – 156 volumes.

Functional Magnetic Resonance Imaging (fMRI) preprocessing

Raw DICOM data images were converted to NIFTI format and organized to conform to the ‘Brain Imaging Data Structure’ specifications (BIDS)(K. J. Gorgolewski et al., 2016). Preprocessing was conducted using FMRIPREP version 1.5.8(Esteban et al., 2019), a Nipype based tool(K. Gorgolewski et al., 2011).

Anatomical Preprocessing

Within the FMRIPREP framework, each T1-weighted (T1w) image was corrected for intensity non-uniformity (INU) using `N4BiasFieldCorrection’ v2.1.0, distributed with `AntsApplyTransforms` (ANTs version 2.2.0). The T1w reference was then skull-stripped with a Nipype implementation of the `antsBrainExtraction.sh` workflow (from ANTs), using OASIS30-ANTs as a target template. Brain tissue segmentation of cerebrospinal fluid (CSF), white matter (WM) and gray matter (GM) was performed on the brain-extracted T1w using `FAST` (FSL version 5.0.9). A T1w-reference map was computed after registration of the INU-corrected T1w image using `mri_robust_template` )FreeSurfer version 6.0.1). Volume-based spatial normalization to one standard space (MNI152NLin2009cAsym) was performed through nonlinear registration with `antsRegistration` tool of ANTs version 2.2.0, using brain-extracted versions of both T1w reference and the T1w template. The ICBM 152 nonlinear Asymmetrical template version 2009 was selected for spatial normalization.

Functional Preprocessing

First, a reference volume and its skull-stripped version were generated using a custom methodology of FMRIPREP, and the susceptibility distortion correction (SDC) was omitted. The BOLD reference was then co-registered to the T1w reference using `MCFLIRT` (FSL version 5.0.9) with the boundary-based registration cost-function. Co-registration was configured with nine degrees of freedom to account for distortions remaining in the BOLD reference. Head-motion parameters with respect to the BOLD reference (transformation matrices, and six corresponding rotation and translation parameters) were estimated before any spatio-temporal filtering. BOLD runs were slice-time corrected using `3dTshift` from AFNI version 16.2.07, and their time-series were resampled onto their original, native space by applying the transforms to correct for head-motion. Several confounding time-series were calculated based on framewise displacement (FD), DVARS and three region-wise global signals (extracted within the CSF, the WM, and the whole-brain masks). Additionally, a set of physiological regressors were extracted to allow for component-based noise correction (CompCor). Principal components were estimated after high-pass filtering of the pre-processed BOLD time-series (using a discrete cosine filter with 128s cut-off) for the two CompCor variants: temporal (tCompCor) and anatomical (aCompCor). Six tCompCor components were then calculated including only the top 5% variable voxels within that subcortical mask. For aCompCor, six components were calculated within the intersection of the subcortical mask and the union of CSF and WM masks calculated in T1w space, after their projection to the native space of each functional run. For each CompCor decomposition, the k components with the largest singular values were retained, sufficient to explain 50% of variance across the nuisance mask. The remaining components were dropped from consideration. All re-samplings was performed with a single interpolation step by composing all the pertinent transformations. Gridded (volumetric) re-samplings were performed using ANTs, configured with Lanczos interpolation to minimize the smoothing effects of other kernels, while non-gridded (surface) re-samplings were performed using `mri_vol2surf` (FreeSurfer). Many internal operations of FMRIPREP use `Nilearn`, principally within the BOLD-processing workflow (for more details of the pipeline, see <https://fmriprep.readthedocs.io/en/stable/workflows.html>).

Eventually, the confounds file in all 1^st^-level fMRI analyses included the following regressors: the time series derived from head motion estimates, their quadratic terms and the temporal derivatives of both series (a total of 24 regressors); the standard deviation of DVARS; the 6 aCompCor components; and framewise displacement (FD). Note that frames that exceeded a threshold of 0.9mm FD were annotated as motion outliers (Siegel et al., 2014). Finally, spatial smoothing of the data was performed using SPM12 (full-width at half-maximum: 6mm). Representational similarity analyses were conducted using non-smoothed data.

Supplementary Results

Supplemental behavioral results

| **Condition** | **Self-referential task** | | | | **Control task** | | | |
| --- | --- | --- | --- | --- | --- | --- | --- | --- |
|  | **Endorsement percentage** | | **RT** | | **Accuracy percentage** | | **RT** | |
|  | ***M*** | ***SD*** | ***M*** | ***SD*** | ***M*** | ***SD*** | ***M*** | ***SD*** |
| High-power | 56.37 | 28.80 | 1.31 | 0.22 | 83.22 | 15.45 | 1.51 | 0.26 |
| Low-power | 33.56 | 22.67 | 1.34 | 0.25 | 86.15 | 14.55 | 1.49 | 0.27 |
| High-affiliation | 90.80 | 14.20 | 1.21 | 0.22 | 84.46 | 14.88 | 1.48 | 0.30 |
| Low-affiliation | 28.83 | 20.24 | 1.27 | 0.20 | 85.59 | 15.50 | 1.49 | 0.26 |

**Table S3. Descriptive statistics of the SRET.** The table depicts means and standard deviations of the percentages of endorsement (for the self-referential conditions) and accuracy (for the control conditions) in the SRET. The corresponding means and standard deviations of response time per conditions are presented as well.

Endorsement of traits in the SRET

| **Effect** | **Beta** | **T-Value** | **P-Value** |
| --- | --- | --- | --- |
| LSAS | -0.01 | -0.14 | 0.89 |
| Intensity | -0.19 | -1.40 | 0.16 |
| Social domain | 0.00 | -0.03 | 0.98 |
| Intensity x Social domain | 0.19 | 1.14 | 0.26 |
| LSAS x Intensity | -0.24 | -1.60 | 0.11 |
| LSAS x Social domain | -0.83 | -5.55 | <2e-16 |
| LSAS x Intensity x Social domain | 0.37 | 2.13 | 0.03 |

**Table S4. Regression results for predicting endorsement percentages in the SRET.** The table presents the regression coefficients (Beta value) of all main and interaction effects, along with their t-value and significance level.

| **Effect** | **Df** | **F-value** | **P-Value** |
| --- | --- | --- | --- |
| Intensity | 1 | 3.46 | 0.06 |
| Social domain | 1 | 58.06 | 3.57E-13 |
| Intensity x Social domain | 1 | 33.37 | 1.95E-08 |

**Table S5. Results from a repeated-measures ANOVA on endorsement percentages in the SRET.**

| **Pairwise comparison** | **Difference** | **95% family-wise  confidence level** | | **P-Value (Tukey HSD)** |
| --- | --- | --- | --- | --- |
|  |  | **Lower** | **Upper** |  |
| Low-affiliation vs. High-affiliation | -19.63 | -29.02 | -10.23 | 8.00E-07 |
| High-power vs. High-affiliation | -34.43 | -43.82 | -25.04 | 0.00E+00 |
| Low-power vs. High-affiliation | -24.36 | -33.75 | -14.97 | 0.00E+00 |
| High-power vs. Low-affiliation | -14.80 | -24.19 | -5.41 | 3.47E-04 |
| Low-power vs. Low-affiliation | -4.74 | -14.13 | 4.65 | 0.56 |
| Low-power vs. High-power | 10.07 | 0.68 | 19.46 | 0.03 |

**Table S6. Differences in endorsement percentages in the SRET.** All pairwise comparisons between conditions in the SRET, that were conducted after finding significant effects of the SRET conditions on endorsement percentages (Table S5). Each pairwise comparison is presented along with the 95% confidence interval of the difference and the significance level of the comparison (Tukey-HSD corrected).

Analysis of response time in the self condition

To examine if there were any effects of the SRET’s conditions and LSAS on response time in the self condition, we conducted a linear moderated regression analysis that was similar to the one depicted in the main text, but with mean response time as the dependent variable. While this regression was significant (R²=.06, Adjusted R²=.04, F(7,288)=2.89, p=.006), none of the main effects or interactions were significant (all ps>.38). However, execution of a repeated-measures ANOVA that did not include LSAS scores on the mean response time in the SRET, did reveal a main effect of social domain on response time (Table S7). This effect was driven by response time in the high-affiliation condition, which were faster than those in both power conditions (Table S8).

| **Effect** | **Df** | **F-value** | **P-Value** |
| --- | --- | --- | --- |
| Intensity | 1 | 2.27 | 0.13 |
| Social domain | 1 | 10.31 | 1.47E-03 |
| Intensity x Social domain | 1 | 0.48 | 0.49 |

**Table S7. Results from a repeated-measures ANOVA on response time during self conditions in the SRET.**

| **Pairwise comparison** | **Difference** | **95% family-wise  confidence level** | | **P-Value (Tukey HSD)** |
| --- | --- | --- | --- | --- |
|  |  | **Lower** | **Upper** |  |
| Low-affiliation vs. High-affiliation | 0.06 | -0.04 | 0.15 | 0.40 |
| High-power vs. High-affiliation | 0.10 | 0.01 | 0.20 | 0.03 |
| Low-power vs. High-affiliation | 0.12 | 0.03 | 0.22 | 0.005 |
| High-power vs. Low-affiliation | 0.04 | -0.05 | 0.14 | 0.62 |
| Low-power vs. Low-affiliation | 0.07 | -0.03 | 0.16 | 0.29 |
| Low-power vs. High-power | 0.02 | -0.07 | 0.12 | 0.94 |

**Table S8. Differences in response time in the SRET during self conditions.** All pairwise comparisons between conditions in the SRET, that were conducted after finding significant effects of the SRET conditions on response time (Table S7). Each pairwise comparison is presented along with the 95% confidence interval of the difference and the significance level of the comparison (Tukey-HSD corrected).

Analysis of accuracy and response time in the control condition

We further applied the above-mentioned regression analysis on both the percentage of correct responses and the mean response time in the control task (i.e. these were two separated analyses). We assumed that accuracy and response time in the control task indicated its difficulty, and we wanted to assure that there were no between-conditions differences in terms of task difficulty; or any interaction effects of LSAS with the SRET’s conditions on accuracy and response time in the control condition.

Thus, we performed a moderated regression analysis on the accuracy percentages and response time in the control condition. The regression model for predicting accuracy in the control task was not significant (R²=.007, Adjusted R²=-.02, F(7,288)=0.30, p=.95), nor was the model for predicting response time (R²=.006, Adjusted R²=-.02, F(7,288)=0.26, p=.97). Note that there were no significant effects of the SRET conditions on either accuracy or response time in the control conditions, also when conducting a repeated-measures ANOVA that did not include LSAS scores on these indices (Table S9).

| **Accuracy** | | | |
| --- | --- | --- | --- |
| **Effect** | **Df** | **F-value** | **P-Value** |
| Intensity | 1 | 1.33 | 0.25 |
| Social domain | 1 | 0.04 | 0.85 |
| Intensity x Social domain | 1 | 0.26 | 0.61 |
| **Response time** | | | |
| **Effect** | **Df** | **F-value** | **P-Value** |
| Intensity | 1 | 0.10 | 0.76 |
| Social domain | 1 | 0.15 | 0.70 |
| Intensity x Social domain | 1 | 0.16 | 0.69 |

**Table S9. Results from a repeated-measures ANOVA on accuracy and response time during control conditions in the SRET.**

Supplemental fMRI results

*
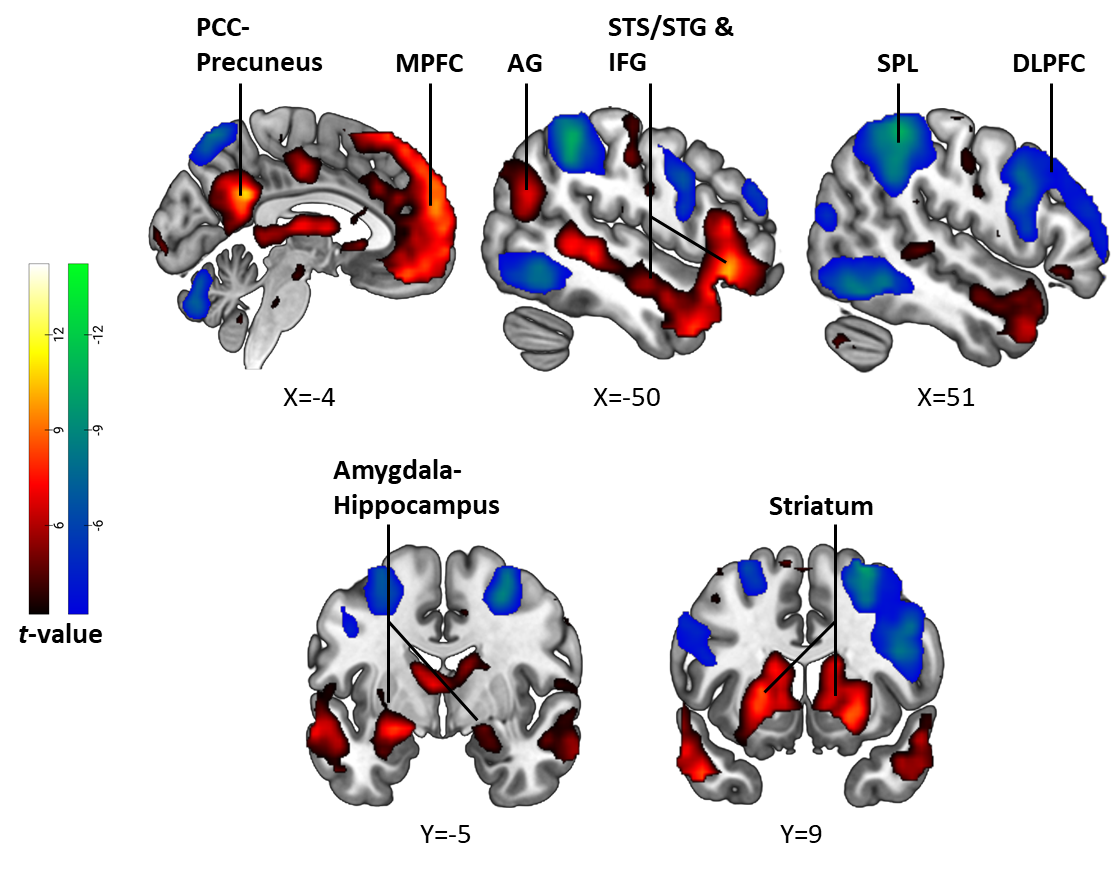
*Brain activation

**Figure S4. Self-referential processing of traits in the SRET.** Statistical parametric maps depicting results from a whole-brain analysis of the Self vs. Control contrast. The red-yellow/blue-green color scaling represents brain activity that was higher in the self/control condition, respectively. The presented brain maps are thresholded at voxel-level p<.001, for display purposes. Notice that the self condition activated classic default-mode network regions that are associated with self-referential processing and social cognition, such as the MPFC and PCC-precuneus, more strongly than the control condition. In contrast, the control condition (determining whether the first two letters of a trait are in alphabetical order) enhanced activity in fronto-parietal regions that are associated with executive functions, such as the DLPFC and SPL, relative to the self condition. Brain images are presented in neurological convention (i.e. right is right). Abbreviations: posterior cingulate cortex (PCC); medial prefrontal cortex (MPFC); (AG); superior temporal sulcus (STS); superior temporal gyrus (STG); superior parietal lobule (SPL); dorsolateral prefrontal cortex (DLPFC).

| **Main effect of social domain: Power > Affiliation** | | | | | | | | |
| --- | --- | --- | --- | --- | --- | --- | --- | --- |
| Brain region | k | Cluster size pFWE | L/R | Peak | Peak | MNI coordinates | | |
|  |  |  |  | t-value | p-value |  |  |  |
|  |  |  |  |  |  | x | y | z |
| Lateral occipital cortex | 60 | 0.021 | R | 4.13 | 0.00E+00 | 30 | -68 | 51 |
|  |  |  | R | 3.75 | 0.00E+00 | 20 | -66 | 51 |
| Lateral occipital cortex, Precuneus | 50 | 0.047 | R | 4.39 | 0.00E+00 | 7 | -77 | 51 |
|  |  |  | R | 4.01 | 0.00E+00 | 16 | -77 | 54 |
| **Main effect of social domain: Affiliation > Power** | | | | | | | | |
| Brain region | k | Cluster size pFWE | L/R | Peak | Peak | MNI coordinates | | |
|  |  |  |  | t-value | p-value |  |  |  |
|  |  |  |  |  |  | x | y | z |
| No significant activations. | | | | | | | | |
| **Main effect of intensity: High > Low** | | | | | | | | |
| Brain region | k | Cluster size pFWE | L/R | Peak | Peak | MNI coordinates | | |
|  |  |  |  | t-value | p-value |  |  |  |
|  |  |  |  |  |  | x | y | z |
| No significant activations. | | | | | | | | |
| **Main effect of intensity: Low > High** | | | | | | | | |
| Brain region | k | Cluster size pFWE | L/R | Peak | Peak | MNI coordinates | | |
|  |  |  |  | t-value | p-value |  |  |  |
|  |  |  |  |  |  | x | y | z |
| VMPFC | 120 | 0.0002 | L | 4.38 | 0.00E+00 | -3 | 55 | -16 |
|  |  |  | R | 4.27 | 0.00E+00 | 7 | 49 | -16 |
|  |  |  | L | 4.02 | 0.00E+00 | 0 | 65 | -10 |
| **Social domain X intensity interaction** | | | | | | | | |
| Brain region | k | Cluster size pFWE | L/R | Peak | Peak | MNI coordinates | | |
|  |  |  |  | t-value | p-value |  |  |  |
|  |  |  |  |  |  | x | y | z |
| Occipital pole | 67 | 0.01 | L | 4.4 | 0.00E+00 | -14 | -94 | -4 |
|  |  |  | L | 3.99 | 0.00E+00 | -21 | -96 | 3 |
| Precentral gyrus, Postcentral gyrus | 54 | 0.03 | R | -3.97 | 0.00E+00 | 2 | -36 | 60 |
|  |  |  | R | -3.8 | 0.00E+00 | 4 | -16 | 60 |
|  |  |  | R | -3.72 | 0.00E+00 | 4 | -25 | 63 |
| Precentral gyrus | 51 | 0.039 | L | -4.4 | 0.00E+00 | -64 | 0 | 15 |
|  |  |  |  | -3.62 | 0.00E+00 | -58 | -5 | 12 |

**Table S10. Effects of social domain and intensity on brain activation in the SRET.** The table presents all clusters arising from whole-brain analyses of the main (group-level) effects of social domain and intensity of traits in the SRET, as well as of the social domain by intensity interaction. Note that the contrast of the interaction was coded as follows: high-power (+1), low-power (-1), high-affiliation (-1), low affiliation (+1). The statistical threshold was set at voxel-level p<.001 and cluster-level pFWE<.05. Coordinates of peak activity are given in MNI space with their peak t-values and corresponding p-values. Abbreviations: Peak in Left/Right hemisphere (L/R); ventromedial prefrontal cortex (VMPFC).

| Brain region | k | Cluster size pFWE | L/R | Peak t-value | Peak  p-value | MNI coordinates | | |
| --- | --- | --- | --- | --- | --- | --- | --- | --- |
|  |  |  |  |  |  | x | y | z |
| Ventral striatum (caudate) | 61 | 0.017 | R | 4.92 | 0.000 | 11 | 21 | -1 |
| mOFC, VMPFC & vACC | 221 | 0.000 | L | 4.16 | 0.000 | -5 | 49 | -19 |
|  |  |  | R | 4.12 | 0.000 | 7 | 51 | -7 |
|  |  |  | R | 3.95 | 0.000 | 9 | 42 | -7 |

**Table S11. Modulation of brain activation by the interaction of social anxiety with traits’ social domain and intensity.** The table presents all clusters arising from a whole-brain regression analysis, wherein we computed the covariance of LSAS scores with a linear contrast of the social domain by intensity interaction in the SRET. The statistical threshold was set at voxel-level p<.001 and cluster-level pFWE<.05. Coordinates of peak activity are given in MNI space with their peak t-values and corresponding p-values. Abbreviations: Peak in Left/Right hemisphere (L/R); medial orbitofrontal cortex (mOFC); ventromedial prefrontal cortex (VMPFC); ventral anterior cingulate cortex (vACC).

Representational Similarity Analysis (RSA)

| **Between-task similarity comparison *in* ROI** | **SRET condition (self>control contrast within condition)** | | | | | | | |  | |  | |
| --- | --- | --- | --- | --- | --- | --- | --- | --- | --- | --- | --- | --- |
|  | **High-power** | | **Low-power** | | **High-affiliation** | | **Low-affiliation** | | **Power**  **(high & low)** | | **Affiliation (high & low)** | |
|  | ***r*** | ***p*** | ***r*** | ***p*** | ***r*** | ***p*** | ***r*** | ***p*** | ***r*** | ***p*** | ***r*** | ***p*** |
| Similarity to incentive salience (monetary reward anticipation) *in* R. vnt. Caudate | 0.33 | 0.02 | -0.13 | 0.34 | 0.09 | 0.52 | 0.00 | 0.99 | 0.17 | 0.24 | 0.10 | 0.48 |

**Table S12. Association of between-task similarity with social anxiety in all conditions.** The table presents values (Pearson’s r coefficients and their corresponding p-values) for the correlation of LSAS with similarity between self-evaluation and incentive salience in the right ventral striatum ROI under all conditions. Abbreviations: left (L); right (R); ventral (vnt.).

Whole-brain RSA

To complement results from the ROI RSA that are presented in the main text, we also conducted a whole-brain RSA between the SRET and the incentive salience contrast from the reward task (i.e. reward anticipation>implicit baseline).

First, we examined whether there were any group-level effects for similarity between self-evaluation in the SRET (i.e. all self >control contrast, across all conditions) and incentive salience. The purpose of this analysis was two-folded: first, we wanted to validate that the RSA indeed yielded meaningful effects by examining the whole-brain level, as a caveat of our design is that we compared between a block-design task (the SRET) and an event-related task (reward) in the RSA. Second, we wanted to examine whether this RSA revealed significant effects of between-task similarity in our ROI, specifically the VS.

To this end, we conducted a whole brain searchlight RSA using non-smoothed data via the RSA toolbox (Nili et al., 2014), while defining Spearman’s rank correlation as the distance measure. The searchlight RSA was performed with a sphere with a radius of 8mm (volume: ~3,200mm³), which covered a radius of about 3-4 voxels around the sphere’s center (Thornton, Weaverdyck, Mildner, & Tamir, 2019). Throughout the process of searchlight RSA, the sphere is centered around each voxel in the brain one step at a time, and that voxel receives the value of similarity between the two contrasts within the sphere. After performing the searchlight, we fisher-z transformed the similarity values in each voxel and submitted them to a 2^nd^-level one-sample t-test via SPM12 in order to determine group-level significance (Axelrod, Rees, & Bar, 2017).

We found significant similarity between self-evaluation in the SRET and incentive salience processing in the reward task in a cluster in the right caudate nucleus (49 voxels at x=14, y=-2, z=18; corrected at voxel-level p<.001 and cluster-level pFWE<.05). Significant between-task similarity was also evident in the bilateral VS, but this reached significance only while using masks that covered this region (left VS: 19 voxels at x=-12, y=7, z=-1; right VS: 10 voxels at x=9, y=10, z=-4; corrected at voxel-level p<.001 and small-volume corrected pFWE<.05). These results are presented in Fig. S5 below. No significant similarity at the whole-brain level was observed between the SRET and the reward responsiveness contrast from the reward task (i.e. winning>losing money).


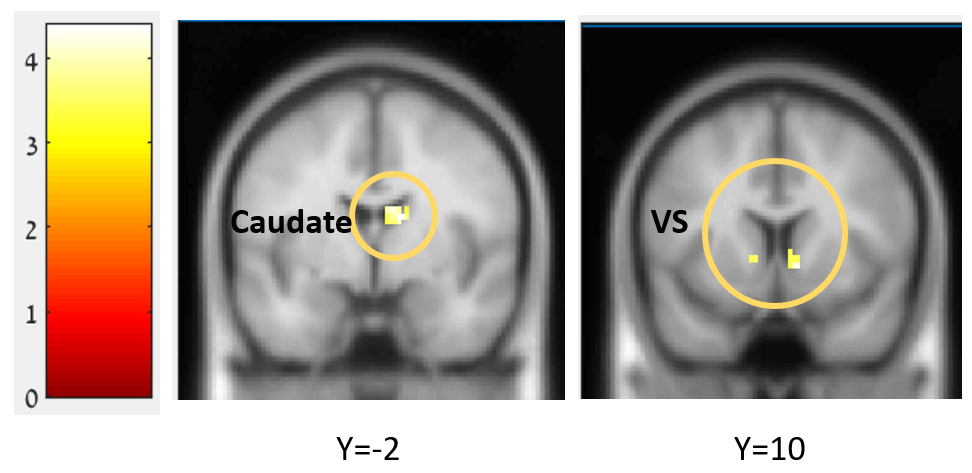


Figure S5. Similarity between self-evaluation and incentive salience. The statistical parametric map depicts results from a searchlight representational similarity analysis between self-evaluation in the SRET (self>control contrast) and incentive salience processing in the monetary reward task (anticipation>baseline contrast). The red-yellow color scaling represents the degree of between-task similarity (t-values). The map is thresholded at voxel-level p<.001 without cluster-extent threshold, for display purposes.

Next, we examined if the correlation that we found between LSAS and similarity of high-power to incentive salience in the ROI RSA within the VS, was also evident at the whole-brain level. To this end, we generated similarity maps for each participant by following the same method described above, but here we focused on similarity of the high-power self>high-power control contrast (SRET) to the anticipation>baseline contrast (reward task). In the 2^nd^-level analysis we conducted a regression analysis in SPM12, where we entered LSAS scores as the covariate of interest. This analysis did not reveal any significant effects at a statistical threshold of voxel-level p<.001 and cluster-level pFWE<.05. However, the correlation of LSAS with between-task similarity was evident at a more lenient threshold of voxel-level p<.01, uncorrected (Fig. S6). This corroborates the results from the ROI RSA results. Specifically, note that in searchlight RSA, each voxel serves as the center of a spherical ROI, as detailed above. Thus, while results from ROI RSA are based on a single selected ROI, the emergence of clusters at the whole-brain level in searchlight RSA means that if several potential ROIs were selected within that region, they would result in similarity effects whose significance level is equal to that found for to ROI we used. Thus, this demonstration of similarity effects at the whole-brain level suggests that the association of LSAS with similarity in the right VS did not only result from the single VS ROI we used; rather, this effect may have been observed for several potential ROIs in the VS. In addition, note that while in the ROI RSA we did not find a significant correlation between LSAS and the between-task similarity indices in the VMPFC, at the whole-brain level this correlations was evident at a statistical threshold of voxel-level p<.001, uncorrected (Fig. S6).


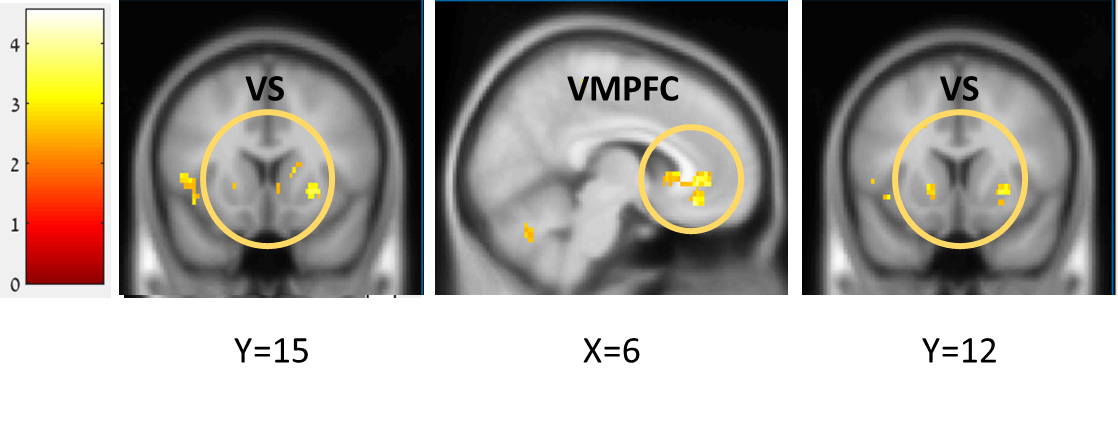


Figure S6. Correlation of social anxiety with similarity between self-evaluation of high-power and incentive salience. The statistical parametric map depicts results from a covariance analysis, testing the correlation of LSAS scores with similarity values yielded by a searchlight representational similarity analysis between self-evaluation of high-power (high-power self>high-power control contrast in the SRET) and incentive salience processing (anticipation>baseline contrast in the monetary reward task). Note that the image on the left contains voxels that are embedded within the right VS ROI that was used for the ROI RSA. The red-yellow color scaling represents the degree of correlation with between-task similarity (t-values). The map is thresholded at voxel-level p<.01 without cluster-extent threshold, for display purposes.

Replication of results while controlling for self-esteem

A important question that arises with regards to the results of this study, is whether they are unique to social anxiety or perhaps may be better explained by individual differences in self-esteem. The main task was a self-evaluation task rather than a social evaluation task, and previous studies showed associations between self-esteem and activation of the VS-VMPC pathway (that was associated here with SA) during self-evaluative tasks (Chavez & Heatherton, 2014; Izuma, Kennedy, Fitzjohn, Sedikides, & Shibata, 2018). To address this issue, we ran all the analyses that yielded significant correlations with LSAS while controlling for self-esteem, as indicated by scores on the Rosenberg Self-Esteem scale (RSE).

We first re-executed the linear moderated regression analysis for predicting endorsement percentages in the SRET. This model included the SRET conditions and LSAS scores as before; and it also included predictors for RSE scores and their interaction with social domain, which was found significant in a separate linear model for predicting endorsement percentages that included only RSE scores and the SRET conditions (β=1.36, t=4.16, p<.001). This regression analysis remained significant (R²=.49, Adjusted R²=.46, F(7,288)=30.66, p<.001), and revealed that the interaction of LSAS with social domain, as well as the 3-way interaction with social domain and intensity, remained significant also while controlling for RSE (Table S13).

| **Effect** | **Beta** | **T-Value** | **P-Value** |
| --- | --- | --- | --- |
| LSAS | 0.001 | 0.013 | 0.989 |
| Social domain | -0.816 | -2.806 | 0.005 |
| Intensity x Social domain | 0.191 | 1.180 | 0.239 |
| LSAS x Intensity | -0.241 | -1.666 | 0.097 |
| LSAS x Social domain | -0.690 | -4.547 | 1.000E-05 |
| LSAS x Intensity x Social domain | 0.369 | 2.209 | 0.028 |
| RSE | 0.032 | 0.491 | 0.624 |
| RSE x Social domain | 0.726 | 3.134 | 0.002 |

**Table S13. Regression results for predicting endorsement percentages in the SRET while controlling for self-esteem.** The table presents the regression coefficients (Beta value) of all main and interaction effects, along with their t-value and significance level.

We next examined if the interaction of LSAS with social domain and intensity of traits in the VS and VMPFC remained significant while controlling for RSE. To this end, we conducted a 2^nd^-level multiple regression analysis on the 1^st^-level contrasts of the interaction effects, which included both LSAS scores and RSE scores as covariates. This analysis revealed that the significant interaction effects in the VS and VMPFC were preserved also while controlling for RSE scores (Table S14).

| Brain region | k | Cluster size pFWE | L/R | Peak t-value | Peak  p-value | MNI coordinates | | |
| --- | --- | --- | --- | --- | --- | --- | --- | --- |
|  |  |  |  |  |  | x | y | z |
| Ventral striatum (caudate) | 48 | 0.049 | R | 5.24 | 0.000 | 11 | 21 | -1 |
| VMPFC | 70 | 0.008 | L | 3.94 | 0.000 | 0 | 44 | -7 |
|  |  |  | R | 3.86 | 0.000 | 7 | 51 | -7 |
|  |  |  | R | 3.62 | 0.000 | 14 | 51 | -1 |

**Table S14. Modulation of brain activation by the interaction of social anxiety with traits’ social domain and intensity while controlling for self-esteem.** The table presents all clusters arising from a whole-brain regression analysis, wherein we computed the covariance of LSAS scores with a linear contrast of the social domain by intensity interaction in the SRET. To control for the effect of self-esteem on brain activity, we also entered a between-subject covariate for RSE scores. The statistical threshold was set at voxel-level p<.001 and cluster-level pFWE<.05. Coordinates of peak activity are given in MNI space with their peak t-values and corresponding p-values. Abbreviations: Peak in Left/Right hemisphere (L/R); ventromedial prefrontal cortex (VMPFC).

Lastly, we tested the correlation of LSAS with neural pattern similarity between high-power and incentive salience in the right VS ROI while controlling for RSE scores, via a partial Pearson correlation. This correlation remained significant as well (r(49)=.31, p=.025). To conclude, this line of analyses indicates that the significant correlations between social anxiety and self-evaluation of high-power found in this study, were indeed unique to SA and were not better explained by trait self-esteem.

References

Anderson, N. H. (1968). Likableness ratings of 555 personality-trait words. *Journal of Personality and Social Psychology*, *9*(3), 272–279. https://doi.org/10.1037/h0025907

Axelrod, V., Rees, G., & Bar, M. (2017). The default network and the combination of cognitive processes that mediate self-generated thought. *Nature Human Behaviour*, *1*(12), 896–910. https://doi.org/10.1038/s41562-017-0244-9

Carlson, J. M., Foti, D., Mujica-Parodi, L. R., Harmon-Jones, E., & Hajcak, G. (2011). Ventral striatal and medial prefrontal BOLD activation is correlated with reward-related electrocortical activity: A combined ERP and fMRI study. *NeuroImage*, *57*(4), 1608–1616. https://doi.org/10.1016/j.neuroimage.2011.05.037

Chavez, R. S., & Heatherton, T. F. (2014). Multimodal frontostriatal connectivity underlies individual differences in self-esteem. *Social Cognitive and Affective Neuroscience*, *10*(3), 364–370.

Erdman, A., Abend, R., Jalon, I., Artzi, M., Gazit, T., Avirame, K., … Harel, E. V. (2020). Ruminative Tendency Relates to Ventral Striatum Functionality: Evidence From Task and Resting-State fMRI. *Frontiers in Psychiatry*, *11*, 67. https://doi.org/10.3389/fpsyt.2020.00067

Esteban, O., Markiewicz, C. J., Blair, R. W., Moodie, C. A., Isik, A. I., Erramuzpe, A., … Gorgolewski, K. J. (2019). fMRIPrep: A robust preprocessing pipeline for functional MRI. *Nature Methods*, *16*(1), 111–116. https://doi.org/10.1038/s41592-018-0235-4

Fisher, P. M., Meltzer, C. C., Ziolko, S. K., Price, J. C., Moses-Kolko, E. L., Berga, S. L., & Hariri, A. R. (2006). Capacity for 5-HT1A–mediated autoregulation predicts amygdala reactivity. *Nature Neuroscience*, *9*(11), 1362–1363. https://doi.org/10.1038/nn1780

Fresco, D. M., Coles, M. E., Heimberg, R. G., Liebowitz, M. R., Hami, S., Stein, M. B., & Goetz, D. (2001). The Liebowitz Social Anxiety Scale: A comparison of the psychometric properties of self-report and clinician-administered formats. *Psychological Medicine*, *31*(06), 1025–1035.

Gilboa-Schechtman, E., Keshet, H., Livne, T., Berger, U., Zabag, R., Hermesh, H., & Marom, S. (2017). Explicit and implicit self-evaluations in social anxiety disorder. *Journal of Abnormal Psychology*, *126*(3), 285–290. https://doi.org/10.1037/abn0000261

Gilead, M., Katzir, M., Eyal, T., & Liberman, N. (2016). Neural correlates of processing “self-conscious” vs. “Basic” emotions. *Neuropsychologia*, *81*, 207–218. https://doi.org/10.1016/j.neuropsychologia.2015.12.009

Gorgolewski, K., Burns, C. D., Madison, C., Clark, D., Halchenko, Y. O., Waskom, M. L., & Ghosh, S. S. (2011). Nipype: A flexible, lightweight and extensible neuroimaging data processing framework in Python. *Frontiers in Neuroinformatics*. https://doi.org/10.3389/fninf.2011.00013

Gorgolewski, K. J., Auer, T., Calhoun, V. D., Craddock, R. C., Das, S., Duff, E. P., … Poldrack, R. A. (2016). The brain imaging data structure, a format for organizing and describing outputs of neuroimaging experiments. *Scientific Data*, *3*(1), 160044. https://doi.org/10.1038/sdata.2016.44

Haines, E. L., & Kray, L. J. (2005). Self-power associations: The possession of power impacts women’s self-concepts. *European Journal of Social Psychology*, *35*(5), 643–662. https://doi.org/10.1002/ejsp.252

Hariri, A. R., Tessitore, A., Mattay, V. S., Fera, F., & Weinberger, D. R. (2002). The amygdala response to emotional stimuli: A comparison of faces and scenes. *Neuroimage*, *17*(1), 317–323.

Izuma, K., Kennedy, K., Fitzjohn, A., Sedikides, C., & Shibata, K. (2018). Neural activity in the reward-related brain regions predicts implicit self-esteem: A novel validity test of psychological measures using neuroimaging. *Journal of Personality and Social Psychology*, *114*(3), 343–357. https://doi.org/10.1037/pspa0000114

Keith Campbell, W., Bosson, J. K., Goheen, T. W., Lakey, C. E., & Kernis, M. H. (2007). Do Narcissists Dislike Themselves “Deep Down Inside”? *Psychological Science*, *18*(3), 227–229. https://doi.org/10.1111/j.1467-9280.2007.01880.x

Lazarov, A., Basel, D., Dolan, S., Dillon, D. G., Pizzagalli, D. A., & Schneier, F. R. (2021). Increased attention allocation to socially threatening faces in social anxiety disorder: A replication study. *Journal of Affective Disorders*, *290*, 169–177. https://doi.org/10.1016/j.jad.2021.04.063

Liebowitz, M. R. (1987). *Social phobia*. Karger Publishers. Retrieved from http://www.karger.com/Article/Abstract/414022

Mennin, D. S., Fresco, D. M., Heimberg, R. G., Schneier, F. R., Davies, S. O., & Liebowitz, M. R. (2002). Screening for social anxiety disorder in the clinical setting: Using the Liebowitz Social Anxiety Scale. *Journal of Anxiety Disorders*, *16*(6), 661–673. https://doi.org/10.1016/S0887-6185(02)00134-2

Nili, H., Wingfield, C., Walther, A., Su, L., Marslen-Wilson, W., & Kriegeskorte, N. (2014). A Toolbox for Representational Similarity Analysis. *PLoS Computational Biology*, *10*(4), e1003553. https://doi.org/10.1371/journal.pcbi.1003553

Raskin, R., & Terry, H. (1988). A principal-components analysis of the Narcissistic Personality Inventory and further evidence of its construct validity. *Journal of Personality and Social Psychology*, *54*(5), 890–902. https://doi.org/10.1037/0022-3514.54.5.890

Rosenberg, M. (1965). Rosenberg self-esteem scale (RSE). *Acceptance and Commitment Therapy. Measures Package*, *61*(52), 18.

Rytwinski, N. K., Fresco, D. M., Heimberg, R. G., Coles, M. E., Liebowitz, M. R., Cissell, S., … Hofmann, S. G. (2009). Screening for social anxiety disorder with the self-report version of the Liebowitz Social Anxiety Scale. *Depression and Anxiety*, *26*(1), 34–38. https://doi.org/10.1002/da.20503

Scult, M. A., Knodt, A. R., Radtke, S. R., Brigidi, B. D., & Hariri, A. R. (2019). Prefrontal Executive Control Rescues Risk for Anxiety Associated with High Threat and Low Reward Brain Function. *Cerebral Cortex*, *29*(1), 70–76. https://doi.org/10.1093/cercor/bhx304

Siegel, J. S., Power, J. D., Dubis, J. W., Vogel, A. C., Church, J. A., Schlaggar, B. L., & Petersen, S. E. (2014). Statistical improvements in functional magnetic resonance imaging analyses produced by censoring high-motion data points: Censoring High Motion Data in fMRI. *Human Brain Mapping*, *35*(5), 1981–1996. https://doi.org/10.1002/hbm.22307

Thornton, M. A., Weaverdyck, M. E., Mildner, J. N., & Tamir, D. I. (2019). People represent their own mental states more distinctly than those of others. *Nature Communications*, *10*(1), 2117. https://doi.org/10.1038/s41467-019-10083-6

von Glischinski, M., Willutzki, U., Stangier, U., Hiller, W., Hoyer, J., Leibing, E., … Hirschfeld, G. (2018). Liebowitz Social Anxiety Scale (LSAS): Optimal cut points for remission and response in a German sample. *Clinical Psychology & Psychotherapy*, *25*(3), 465–473. https://doi.org/10.1002/cpp.2179

Wiggins, J. S. (1979). A psychological taxonomy of trait-descriptive terms: The interpersonal domain. *Journal of Personality and Social Psychology*, *37*(3), 395–412. https://doi.org/10.1037/0022-3514.37.3.395

Wiggins, J. S., & Broughton, R. (1991). A geometric taxonomy of personality scales. *European Journal of Personality*, *5*(5), 343–365. https://doi.org/10.1002/per.2410050503

Wojciszke, B., & Sobiczewska, P. (2013). Memory and Self-Esteem: The Role of Agentic and Communal Content. *Social Psychology*, *44*(2), 95–103. https://doi.org/10.1027/1864-9335/a000149

Zhou, Z., Zhu, G., Hariri, A. R., Enoch, M.-A., Scott, D., Sinha, R., … Goldman, D. (2008). Genetic variation in human NPY expression affects stress response and emotion. *Nature*, *452*(7190), 997–1001. https://doi.org/10.1038/nature06858
